# CEP192 is a novel prognostic marker and correlates with the immune microenvironment in hepatocellular carcinoma

**DOI:** 10.3389/fimmu.2022.950884

**Published:** 2022-09-27

**Authors:** Yanli Liu, Wanmei Liang, Yabin Chang, Zehui He, Meijian Wu, Haozhi Zheng, Xinrong Ke, Minjia Lv, Qingqian Liu, Qinyu Liu, Waner Tang, Qiaoling Huang, Yu Lu, Min He, Qijun Yang, Chunpan Mo, Jiefan Wang, Kunwei Peng, Zhiqun Min, Hang Su, Jingqi Chen

**Affiliations:** ^1^ Guangzhou Key Laboratory for Research and Development of Nano-Biomedical Technology for Diagnosis and Therapy and Guangdong Provincial Education Department Key Laboratory of Nano-Immunoregulation Tumour Microenvironment, Department of Oncology and Translational Medicine Center, The Second Affiliated Hospital, Guangzhou Medical University, Guangzhou, China; ^2^ Department of Gynecology, The Second Affiliated Hospital, Guangzhou Medical University, Guangzhou, China; ^3^ The Second Clinical Medical School, Guangzhou Medical University, Guangzhou, China; ^4^ Central Laboratory, The Second Affiliated Hospital, Guangzhou Medical University, Guangzhou, China

**Keywords:** hepatocellular carcinoma, CEP192, cell cycle, cancer stem cell, immune suppression

## Abstract

Hepatocellular carcinoma (HCC) responds poorly to standard chemotherapy or targeted therapy; hence, exploration for novel therapeutic targets is urgently needed. CEP192 protein is indispensable for centrosome amplification, which has been extensively characterized in both hematological malignancies and solid tumors. Here, we combined bioinformatics and experimental approaches to assess the potential of CEP192 as a prognostic and therapeutic target in HCC. CEP192 expression increased with tumor stage and was associated with poor clinicopathologic features, frequent recurrence, and higher mortality. Upon single-cell RNA sequencing, CEP192 was found to be involved in the proliferation and self-renewal of hepatic progenitor-like cells. This observation was further evidenced using CEP192 silencing, which prevented tumor cell proliferation and self-renewal by arresting cells in the G0/G1 phase of the cell cycle. Notably, CEP192 was highly correlated with multiple tumor-associated cytokine ligand–receptor axes, including IL11–IL11RA, IL6–IL6R, and IL13–IL13RA1, which could promote interactions between hepatic progenitor-like cells, PLVAP+ endothelial cells, tumor-associated macrophages, and CD4+ T cells. Consequently, CEP192 expression was closely associated with an immunosuppressive tumor microenvironment and low immunophenoscores, making it a potential predictor of response to immune checkpoint inhibitors. Taken together, our results unravel a novel onco-immunological role of CEP192 in establishing the immunosuppressive tumor microenvironment and provide a novel biomarker, as well as a potential target for therapeutic intervention of HCC.

## Introduction

According to Global Cancer Statistics 2020 produced by the International Agency for Research on Cancer, primary liver cancer is the sixth most common incident cancer and the third leading cause of cancer-related mortality worldwide, with about 906,000 new cases and 830,000 deaths (1). Hepatocellular carcinoma (HCC), accounting for approximately 75%–85% of primary liver cancers, has an extremely poor prognosis with a mortality rate almost equal to the incidence rate and a 5-year overall survival (OS) rate below 12% (2–4). Evidence suggests that early diagnosis and prompt treatment could improve long-term outcomes; however, HCC is typically diagnosed at advanced stages where liver function is compromised and treatment options are limited (5). Thus, effective strategies for diagnosis, prognostication, and treatment of HCC remain urgently needed.

Surveillance using abdominal ultrasonography and serum alpha-fetoprotein (AFP) was recommended in high-risk populations to detect HCC early for potentially curative surgical resection or transplantation (6). However, the accuracy of surveillance is relatively low (40%–50%), and approximately half of the patients are diagnosed with advanced stages when systemic therapy remains the only standard treatment option. Notably, in the last decades, the landscape of systemic therapeutic options has considerably expanded, with the recent impressive advances in molecular-targeted therapies and immunotherapies (5). Immune checkpoint inhibitors (ICIs), with good tolerable safety profiles and durable antineoplastic effects in a broad spectrum of malignancies, have been the focus of a surge in clinical trials in combination with other immunomodulators or conventional systemic anticancer therapies, including molecular-targeted therapy or chemotherapy (7). Both preclinical studies and clinical trials have shown that antiangiogenic agents could sensitize patients to ICIs *via* reprogramming the tumor microenvironment, leading to the approval of bevacizumab (anti-VEGFA) plus atezolizumab or sintilimab (anti-PD-L1/PD-1) in the first-line settings, based on the superior OS and progression-free survival (PFS) versus current standard care (sorafenib) for patients with unresectable HCC (8–11). However, objective response rates (ORR) of these regimens remain low in the order of 10%–50% (7). Therefore, discovering and validating predictive biomarkers of outcome and response to immunotherapy can assist in selecting patients who may derive the greatest therapeutic benefit.

Tumor aneuploidy, alone or in combination with tumor mutation burden (TMB), is a biomarker of response to ICIs. It is noteworthy that tumor aneuploidy was positively correlated with immune evasion markers and negatively correlated with cytotoxic immune cell infiltration (12). Centrosome abnormalities and amplification are frequently observed in cancer cells and are closely linked to genomic instabilities, which may serve as a mechanism for aneuploidy development (13). A recent study suggested that targeting Polo-like kinase 4 (PLK4), a centrosome duplication regulator, could suppress tumor proliferation *via* inhibiting the cell cycle and eliciting anti-tumor immunity, with durable effect even in late-stage mouse HCC (14). PLK4 appears to be the center of centriole duplication, and CEP192, a centrosomal scaffold protein localized to nearly the entire length of mother and daughter centrioles, is indispensable for PLK4-mediated centriole duplication through binding and recruiting of PLK4 to centrosomes (15). In recent years, CEP192 has been identified as a new gene in the progression of non-alcoholic fatty liver disease (NAFLD) to HCC (16). Nevertheless, until recently, still only very little is known about the carcinogenic mechanism of CEP192 in tumor progression.

Here, to explore the role and underlying mechanisms of CEP192 in HCC progression, we performed large-scale bioinformatic analyses of 2,151 samples from the TCGA, ICGC, and GEO liver cancer datasets. We found that increased expression of CEP192 was associated with advanced HCC stage and poor survival, suggesting an oncogenic function of CEP192. The tumor-promoting effect of CEP192 was subsequently confirmed using the siRNA silencing experiment *in vitro*. Importantly, based on single-cell RNA sequencing data, we observed an abundant expression of CEP192 in hepatic progenitor-like cells, which could respond to hypoxia and secrete high levels of VEGFA. Moreover, the HPC-like cells, PLVAP+ ECs, TAMs, and CD4+ T cells of patients with high levels of CEP192 may interact through various cytokine ligand–receptor axes, including IL11–IL11RA, IL6–IL6R, and IL13-IL13RA, perhaps contributing to an immunosuppressive ecosystem. Taken together, our work reveals a novel function of CEP192 in HCC progression.

## Materials and methods

### mRNA expression analysis of CEP192 gene in different HCC datasets

To analyze the expression difference of CEP192 between HCC tumor tissues and adjacent non-tumor tissues, a total of 8 HCC messenger RNA (mRNA) expression datasets were downloaded from The Cancer Genome Atlas (TCGA) and International Cancer Genome Consortium (ICGC) database as well as Gene Expression Omnibus (GEO) datasets (GSE14520, GSE45267, GSE121248, GSE36376, GSE76427, and GSE65372). Detailed information of these datasets is listed in **Supplementary Table S1**. Graphical presentation and statistical analyses of the CEP192 expression were conducted using GraphPad Prism v 8.0 (17).

### Correlation analysis of CEP192 and clinicopathological characteristics

GSE89377 (including 13 paired nontumor samples, 32 chronic hepatitis/cirrhosis samples, 22 samples with dysplastic nodules, 14 HCC samples with T1 stage, and 26 HCC samples with T2 and T3 stage, https://www.ncbi.nlm.nih.gov/geo/query/acc.cgi?acc=GSE89377) was downloaded to examine CEP192 expression in preneoplastic lesions and HCC tissues. Moreover, clinical information of TCGA-HCC was downloaded to assess the correlation of CEP192 with HCC patients’ clinicopathologic characteristics (https://portal.gdc.cancer.gov/). The association between CEP192 expression and clinicopathological variables, including T stage, N stage, M stage, pathologic stage, tumor status, gender, race, age, weight, height, BMI, residual tumor, histologic grade, adjacent hepatic tissue inflammation, AFP (ng/ml), albumin (g/dl), prothrombin time, Child-Pugh grade, fibrosis Ishak score, vascular invasion, OS event, disease-free survival (DFS) event, and PFS event, was estimated *via* a Chi-square or a Fisher’s exact test. All variables with a *p*-value less than 0.05 in Chi-square or Fisher’s exact test were included in the univariate logistic regression model to infer the odds ratio for each variable. Additionally, Kaplan–Meier survival estimate and log-rank test were conducted to evaluate the impact of CEP192 on OS, DSS, and PFS.

### Construction of prognostic nomogram

To provide a clinically quantitative tool for predicting the survival outcome of HCC patients, a nomogram based on clinical risk factors was necessary. Firstly, the univariate Cox proportional hazard model was used to assess the hazard ratios (HRs) of CEP192 expression and some clinicopathological variables (T stage, N stage, M stage, tumor status, pathologic stage, gender, age, histologic grade, AFP, and vascular invasion) on OS, DSS, and PFS of HCC patients. Furthermore, the variables with significance (*p*-value < 0.1) in univariable analysis were subjected to multivariate Cox regression analysis. Meanwhile, independent risk variables based on the univariate model were integrated to construct a nomogram. Secondly, receiver operating characteristic (ROC) curves were plotted and the areas under the ROC curves (AUCs) were also calculated using the package “timeROC” to test the specificity and sensitivity of the nomogram in predicting 1-year, 3-year, and 5-year survival probabilities. Thirdly, calibration curves of the nomogram were created to measure the agreement of the predicted and observed survival. Moreover, decision curve analysis (DCA) was carried out to evaluate the clinical performance of the prognostic nomogram.

### Immunohistochemical analysis of CEP192 expression and CD8^+^ T-cell infiltration level in HCC tissues and normal tissues

Fifteen paraffin-embedded HCC tissues and adjacent non-tumor tissue were collected for immunohistochemistry (IHC) staining. Each tissue block was serially sectioned (4 μm) onto glass slides. These sections were baked (65°C, 1 h), deparaffinized in xylene, and rehydrated in a sequential ethanol gradient (from 95% to 50%). Subsequently, antigen retrieval was performed in boiling sodium citrate buffer (10 mM, pH 6.0, BOSTER, 16H10A24) for 10 min and endogenous peroxidase activity was blocked with a blocking buffer (Reagent 1, MXB Biotechnologies, KIT-9720) for 10 min, followed by nonspecial staining blocking (Reagent 2, MXB Biotechnologies, KIT-9720) for 15 min at room temperature. The primary antibody for CEP192 (1:200 dilution, #18832-1-AP; Proteintech) and CD8 (1:1,000 dilution, #66868-1-Ig, Proteintech) was incubated overnight at 4°C. After washing thrice with phosphate-buffered saline solution (PBS), biotinylated goat anti-mouse/rabbit antibody (Reagent 3, MXB Biotechnologies, KIT-9720) was added for 10 min, and then streptavidin peroxidase (Reagent 4, MXB Biotechnologies, KIT-9720) was applied for 10 min. Lastly, the cells were visualized with 3,3′-diaminobenzidine (DAB, MXB Biotechnologies, DAB-0031), counterstained with hematoxylin (abs9217, absin), dehydrated, and mounted. The IHC staining results were captured through laser microdissection microscopes (Leica, LMD6, German) and evaluated using ImageJ-IHC Profiler software (18). In brief, the staining intensity (0, negative; 1, weakly positive; 2, positive; 3, high positive) and the proportion of stained cells (0, 0%; 1, <10%; 2, 11%–50%; 3, 51%–80%; 4, >80%) were calculated and multiplied to provide an IHC score for CEP192 expression.

### Cell culture and small interfering RNA transfection

The liver HCC cell lines Hep3B and SK-Hep1 (ATCC) were cultured in Dulbecco’s modified Eagle’s medium (DMEM) supplemented with 10% fetal bovine serum (FBS) at 37°C in a 5% CO_2_ humidified incubator. Cells were seeded at ~40% confluency and transfected with small interfering RNA (siRNA) after 18–24 h using the riboFECT CP Transfection Kit (C10511-05, RIBOBIO Biotechnology) according to the manufacturer’s protocol. The siRNA duplexes for negative control (NC) (forward: 5′-UUCUCCGAACGUGUCACGUdTdT3′; reverse: 5′-ACGUGACACGUUCGGAGAAdTdT-3′), CEP192-siRNA#1 (forward: 5′-CCAGGAGCCUAUAGAUGAAdTdT-3′; reverse: 5′-UUCAUCUAUAGGCUCCUGGdTdT-3′), and CEP192-siRNA#2 (forward: 5′-GAUGCCAUUUGGUCACCAAdTdT-3′; reverse: 5′-UUGGUGACCAAAUGGCACdTdT-3′) were synthesized in RIBOBIO Biotechnology (Guangzhou, China).

### Real-time quantitative reverse transcription polymerase chain reaction

On day 3 post-transfection, total RNA was extracted from the transfected cells using the RNAeasy™ RNA Isolation Kit (R0024, Beyotime) according to the manufacturer’s instructions. Reverse transcription was completed with RT primer mix (oligo dT primer and random 6 mers) using the PrimeScriptTM RT reagent Kit (RR047A, Takara). Quantitative real-time RT-PCR (qRT-PCR) was implemented on a QuantStudio 7 Flex system (Applied Biosystems, USA) with TB Green Premix Ex Taq (RR820A, TaKaRa, Japan). Primers designed with Primer-BLAST (National Center for Biotechnology Information, NCBI) were as follows: human CEP192, forward: 5’-CACTTGCTAGGGATAGATCCAGC-3’ and reverse: 5’-ACCCGGATGGAACTGAAAATC-3’; human GAPDH, forward: 5’-ACAACTTTGGTATCGTGGAAGG-3’ and reverse: 5’-GCCATCACGCCACAGTTTC-3’. Relative quantification was computed using the 2^−ΔΔCT^ method. The experiments were performed in triplicate.

### Western blotting

On day 3 post-transfection, total protein was extracted from the transfected cells using cell lysis buffer (P0013, Beyotime) with PMSF (ST506, Beyotime) and protease phosphatase inhibitor cocktail (P1046, Beyotime), followed by BCA assay for protein quantification (P001, Beyotime). Protein lysates in InstantView™ SEMS-PAGE protein staining and loading buffer (Beyotime, P0280) were loaded on 10% sodium dodecyl sulfate-polyacrylamide (SEMS-PAGE) gels and then transferred to polyvinylidene difluoride (PVDF) membranes (cat#1620177, Bio-Rad), blocked with 5% skimmed milk, and incubated with primary antibodies for CEP192 (1:1,000; Bethyl, A302–324A) and GAPDH (1:1,000; #8884, Cell Signaling Technology) overnight at 4°C. The membranes were washed and incubated with horse anti-rabbit IgG coupled with horseradish peroxidase (HRP) (1:10,000; #7076, Cell Signaling Technology) for 1 h, then developed with SuperSignal TM West Pico PLUS Chemiluminescent Substrate (WB322159, Thermo). Images were acquired with ChemisScope 6100 Touch (Clinx, China).

### Immunofluorescence staining

The SK-Hep1 cells were grown on sterile coverslips and then fixed with 4% paraformaldehyde. After blocking with Intercept^®^ Blocking Buffer (927-70001, LI-COR, USA), cells were incubated with primary antibodies against CEP192 (1:200, 18832-1-AP, Proteintech, China) and γ-tubulin (1:200, 66320-1-AP, Proteintech, China), followed by incubation with Alexa Fluor 488-conjugated goat anti-rabbit IgG (1: 200, 2112, EMAR, China) and CY3-conjugated goat anti-mouse IgG (1:200, S0013, Affinity, US). Finally, cells were stained with 4,6-diamidino-2-phenylindole (DAPI, 20180627, Solarbio, China), and images were captured using a laser scanning confocal microscope (LMD6, LEICA, Germany).

### Cell proliferation and colony formation assays

Seven days post-transfection, cell viability was measured using MTT [3-(4,5-dimethylthiazol-2-yl)-2,5-diphenyl tetrazolium bromide], which is metabolized to formazan crystals in live cells. This metabolite could be dissolved in dimethyl sulfoxide (DMSO) and measured at 570 nm in a microtiter plate reader (Berthold-LB943). Cell proliferation and self-renewal ability were further detected through colony formation assay. Briefly, after transfection with CEP192 or NC siRNAs, 300 cells were seeded into each well of a 12-well plate and incubated at 37°C for 14 days in a 5% CO_2_ atmosphere. Then, colonies were fixed with 4% paraformaldehyde, stained with 0.1% crystal violet, and counted with ImageJ software.

### Cell cycle analysis

After 7 days, the transfected cells were collected, washed once with PBS, and fixed with precooled 70% ethanol at 4°C overnight. Subsequently, ethanol was removed and cells were washed twice with PBS and stained with propidium iodide (PI)/RNase A buffer (ST511, Beyotime) for 30 min in the dark. Then, the cell cycle distribution was detected using flow cytometry (Quantenon 4025, Agilent).

### Association analysis of CEP192 and tumor-infiltrating immune cells

The association between CEP192 expression and the abundance of tumor-infiltrating immune cells was estimated using data downloaded from Tumor and Immune System Interaction Database (TISIDB, http://cis.hku.hk/TISIDB/) (19) and Tumor IMmune Estimation Resource (TIMER, http://www.cistrome.shinyapps.io).

### Correlation analysis of CEP192 and immune checkpoints

ICI therapy against CTLA-4 or PD-1/PD-L1 has achieved remarkable clinical outcomes and revolutionized cancer treatment, but not all patients respond to this therapy. Therefore, reliable biomarkers to predict response to ICIs are urgently needed. Tumor-mutation burden (TMB) and immunophenoscore (IPS) have been reported as potential predictive biomarkers in patients receiving ICIs (20). To explore whether CEP192 was a predictive biomarker for ICI response, the correlation of CEP192 with TMB and IPS score was examined using data derived from The Cancer Immunome Atlas (TCIA) (https://tcia.at/home).

### Single-cell RNA sequencing analysis

Raw ScRNA-seq data from 19 liver cancer samples (GSM4050085, GSM4050086, GSM4050087, GSM4050088, GSM4050089, GSM4050090, GSM4050091, GSM4050092, GSM4050093, GSM4050094, GSM4050095, GSM4050096, GSM4050098, GSM4050100, GSM4050102, GSM4050104, GSM4050106, GSM4050108, and GSM4050110) was downloaded from Gene Expression Omnibus (GEO) (dataset GSE125449) (https://www.ncbi.nlm.nih.gov/geo/query/acc.cgi?acc=GSE125449). R package Seurat V4.0 was used to process the single-cell data expression matrix (21). At first, cells with <50 genes and >5% mitochondrial gene proportion were excluded from analysis. After normalizing the scRNA-seq data using the LogNormalize method, 1,500 highly variable genes (HVGs) were identified using the “FindVariableGenes” function. Then, dimensionality reduction was obtained using principal component analysis (PCA). Next, the K-nearest neighbor was automatically weighted with “FindNeighbors” and cells were combined in a highest resolution (resolution = 1) with “FindClusters”. Afterward, these clusters were visualized using “UMAP” and annotated according to the marker genes reported previously (22). Single-cell pseudotime trajectories of the PLVAP+ endothelial cells (PLVAP+ ECs) were reconstructed and analyzed with the package “Monocle” (23).

### Statistical analysis

For significance analysis, normality and homoscedasticity assumptions are tested for each variable first. Unpaired or paired *t*-test was employed to assess the expression difference of CEP192 between HCC tumor tissues and adjacent non-tumor tissues. Mann–Whitney *U* test (Wilcoxon rank-sum test) was used to evaluate the abundance differences of tumor-infiltrated immune cells/genes between CEP192 high and CEP192 low groups. The distinct CEP192 groups were divided using the median expression of CEP192 in 836 HCC patients, which was calculated with the function “median” in R. Moreover, Chi-square or Fisher’s exact test was applied for inferring the association between CEP192 expression and clinicopathological variables. A univariate logistic regression model was also adopted to calculate the odds ratio for each variable. As for prognosis analysis, the Kaplan–Meier method was used to estimate survival probabilities and the log-rank test was conducted to determine the differences in the survival curves. Univariate or multivariate analyses with the Cox proportional hazards regression model, along with hazard ratios (HRs) and 95% confidence intervals (CIs), were performed to search for risk factors for survival. In addition, one-way or two-way ANOVA multiple comparisons were carried out to compare the differences in cell biological characteristics between the control group and the CEP192-knockdown group. Statistical significance was shown with asterisks (*p < 0.05; **p < 0.01; ***p < 0.001; ****p < 0.0001).

## Results

### Increased CEP192 expression associated with advanced clinicopathological characteristics in HCC patients

**Figure 1:**
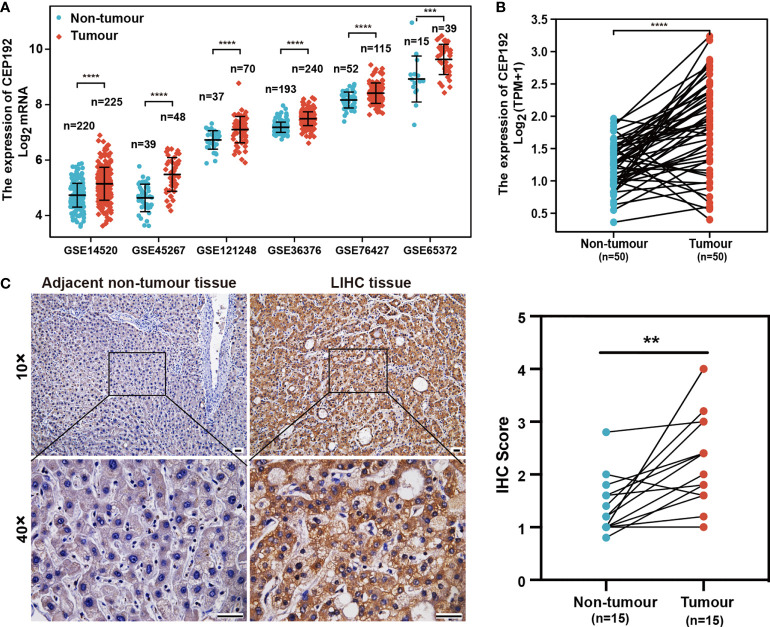
CEP192 was overexpressed in human HCC tissues. **(A)** CEP192 was highly expressed in human HCC tissues (T) versus adjacent non-tumor tissues (N) based on the RNA-seq data from the GEO database including GSE14520 (T, *n* = 225; N, *n* = 220; mean ± SEM, Mann–Whitney test), GSE45267 (T, *n* = 48; N, *n* = 39; mean ± SEM, *t*-test), GSE121248 (T, *n* = 70; N, *n* = 37; mean ± SEM, Mann–Whitney test), GSE36376 (T, *n* = 240; N, *n* = 193; mean ± SEM, Mann–Whitney test), GSE76427 (T, *n* = 115; N, *n* = 52; mean ± SEM, *t*-test), and GSE65372 (T, *n* = 39; N, *n* = 15; mean ± SEM, *t*-test); ***p < 0.001, ****p < 0.0001. **(B)** CEP192 expression was increased in 50 HCC samples compared to their matched non-tumor liver samples according to the RNA-seq data analysis of TCGA; paired *t*-test, ****p < 0.0001. **(C)** Representative IHC images demonstrated higher levels of CEP192 in HCC tumor tissues compared with adjacent non-tumor liver tissues (scale bar: 200 μm). The IHC images were quantified with an IHC score using ImageJ (*n* = 15); paired *t*-test, **p < 0.01.

To evaluate the potential role of CEP192 in HCC, we first examined CEP192 mRNA expression in GEO, TCGA, and ICGC databases. As shown in **Figure 1A** and **Figure S1A**, CEP192 was significantly increased in HCC tissues compared with that in adjacent non-tumor tissues based on seven HCC datasets, namely, GSE14520 (*p* < 0.001), GSE45267 (*p* < 0.001), GSE121248 (*p* < 0.001), GSE36376 (*p* < 0.001), GSE76427 (*p* < 0.001), GSE65372 (*p* < 0.001), and ICGC (*p* < 0.001). Moreover, CEP192 was found to be upregulated in HCC tissues compared with that in the paired adjacent non-tumor tissues (TCGA, *p* < 0.001) (**Figure 1B**). In addition, CEP192 was increased not only in HCC but also in other types of cancer, including cholangiocarcinoma, esophageal carcinoma, head and neck squamous cell carcinoma, and lung adenocarcinoma (**Figure S1B**). Furthermore, 15 clinical tissues of HCC were collected to verify the protein expression levels of CEP192 using IHC analysis, and the results revealed a higher level of the CEP192 protein in HCC tissues than that in adjacent non-tumor tissues (**Figure 1C**). Taken together, these results indicate that CEP192 was significantly increased in HCC tissues.

**Figure 2:**
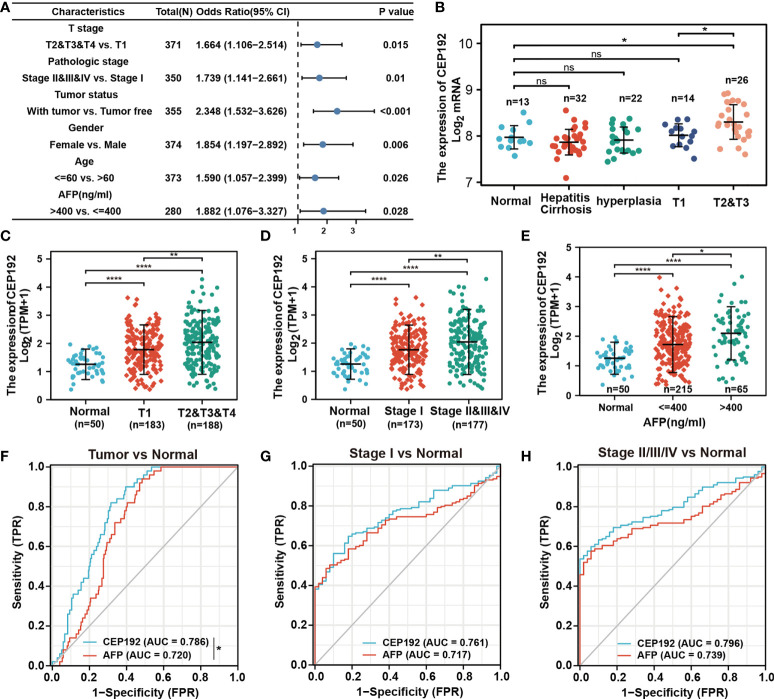
CEP192 was correlated with advanced clinicopathological features of HCC patients. **(A)** CEP192 was shown to be highly associated with the T stage (*n* = 371), pathologic stage (*n* = 350), tumor status (*n* = 355), gender (*n* = 374), age (*n* = 373), and AFP secretion level (*n* = 280); generalized linear model (GLM). **(B)** The expression levels of CEP192 in precancerous lesions including hepatitis (*n* = 13), cirrhosis (*n* = 32), and hyperplasia (*n* = 22), as well as in HCC tissues at different T stages (T1, *n* = 14; T2&T3, *n* = 26) (GSE89377); mean ± SEM, ordinary one-way ANOVA, *p < 0.05. **(C)** CEP192 gene expression (RNA-seq) of TCGA-LIHC samples at different T stages of liver cancer (Normal, *n* = 50; T1, *n* = 183; T2&T3&T4, *n* = 188); mean ± SEM, ordinary one-way ANOVA, **p < 0.01, ****p < 0.0001. **(D)** CEP192 gene expression (RNA-seq) of TCGA-LIHC samples at different pathologic stages of liver cancer (Normal, *n* = 50; Stage I, *n* = 173; Stage II&III&IV, *n* = 177); mean ± SEM, ordinary one-way ANOVA, **p < 0.01, ****p < 0.0001. **(E)** CEP192 gene expression (RNA-seq) from TCGA-LIHC samples with different AFP secretion levels [Normal, *n* = 50; AFP (ng/ml) ≤ 400, *n* = 215; AFP (ng/ml) > 400, *n* = 65]; mean ± SEM, ordinary one-way ANOVA, *p < 0.05, ****p < 0.0001. **(F–H)** Receiver operating characteristic (ROC) curves and the area under the ROC curve (AUC) were used to evaluate the sensitivity and specificity of CEP192 expression in diagnostic performance of HCC (DeLong’s test, *p = 0.049) **(F)**, early-stage HCC **(G)**, and late-stage HCC **(H)**. NS, non significant.

The relationship between CEP192 expression and clinicopathological variables of HCC patients was further studied on 371 patients from the TCGA-LIHC dataset. Chi-square test and Fisher exact test illustrated a significant correlation of CEP192 expression levels with pathologic stage (*p* = 0.008), tumor status (*p* < 0.001), sex (*p* = 0.008), age (*p* = 0.034), AFP expression (*p* = 0.038), DFS event (*p* < 0.001), and PFS event (*p* = 0.007) of HCC patients (**Table 1**). Moreover, logistic regression (**Figure 2A**) and one-way ANOVA (**Figures 2B** **–E**) indicated that high CEP192 expression was notably associated with high T classification (T2/T3/T4 vs.T1, **Figures 2B, C**), advanced pathologic stage (II/III/IV vs. I, **Figure 2D**), and high AFP expression level (**Figure 2E**). In contrast, there was no significant relevance of CEP192 expression to precancerous lesions, such as fibrosis and cirrhosis (**Table 1**, **Figure 2B**). These results give a clue that CEP192 may be a specific biomarker for HCC. Therefore, the diagnostic ability of CEP192 in HCC patients was assessed using an AUC. As shown in **Figure 2F**, CEP192 had a slightly better AUC score than the gold standard diagnostic marker AFP (0.786 vs. 0.720, *p* < 0.05, DeLong’s test). Notably, CEP192 performed better for predicting advanced-stage HCC patients compared to those in early-stage HCC (AUC: 0.796 vs. 0.761, **Figures 2G, H**). These data indicated that CEP192 could be a potential diagnostic biomarker for HCC, especially for advanced-stage HCC.

**Table 1:** Correlations between CEP192 expression and clinical characteristics of HCC patients.

Characteristic	Levels	Low expression of CEP192	High expression of CEP192	*p*	Statistic	Method
*n*		187	187			
T stage, *n* (%)	T1	103 (56.3%)	80 (43.7%)	0.083	6.68	*χ* ^2^ test
	T2	44 (46.3%)	51 (53.7%)			
	T3	32 (40%)	48 (60%)			
	T4	6 (46.2%)	7 (53.8%)			
N stage, *n* (%)	N0	127 (50%)	127 (50%)	0.622		Fisher test
	N1	1 (25%)	3 (75%)			
M stage, *n* (%)	M0	137 (51.1%)	131 (48.9%)	0.623		Fisher test
	M1	3 (75%)	1 (25%)			
Pathologic stage, *n* (%)	Stage I	100 (57.8%)	73 (42.2%)	**0.008**		Fisher test
	Stage II	42 (48.3%)	45 (51.7%)			
	Stage III	32 (37.6%)	53 (62.4%)			
	Stage IV	4 (80%)	1 (20%)			
Tumor status, *n* (%)	Tumor free	119 (58.9%)	83 (41.1%)	**<0.001**	14.53	*χ* ^2^ test
	With tumor	58 (37.9%)	95 (62.1%)			
Gender, *n* (%)	Female	48 (39.7%)	73 (60.3%)	**0.008**	7.04	*χ* ^2^ test
	Male	139 (54.9%)	114 (45.1%)			
Race, *n* (%)	Asian	82 (51.2%)	78 (48.8%)	0.641	0.89	*χ* ^2^ test
	Black or African American	7 (41.2%)	10 (58.8%)			
	White	88 (47.6%)	97 (52.4%)			
Age, *n* (%)	≤60	78 (44.1%)	99 (55.9%)	**0.034**	4.51	*χ* ^2^ test
	>60	109 (55.6%)	87 (44.4%)			
Weight, *n* (%)	≤70	89 (48.4%)	95 (51.6%)	0.220	1.51	*χ* ^2^ test
	>70	90 (55.6%)	72 (44.4%)			
Height, *n* (%)	<170	105 (52.2%)	96 (47.8%)	0.670	0.18	*χ* ^2^ test
	≥170	69 (49.3%)	71 (50.7%)			
BMI, *n* (%)	≤25	86 (48.6%)	91 (51.4%)	0.341	0.91	*χ* ^2^ test
	>25	87 (54.4%)	73 (45.6%)			
Residual tumor, *n* (%)	R0	168 (51.4%)	159 (48.6%)	0.462		Fisher test
	R1	7 (41.2%)	10 (58.8%)			
	R2	1 (100%)	0 (0%)			
Histologic grade, *n* (%)	G1	33 (60%)	22 (40%)	0.284	3.8	*χ* ^2^ test
	G2	90 (50.6%)	88 (49.4%)			
	G3	55 (44.4%)	69 (55.6%)			
	G4	6 (50%)	6 (50%)			
Adjacent hepatic tissue inflammation, *n* (%)	None	69 (58.5%)	49 (41.5%)	0.348	2.11	*χ* ^2^ test
	Mild	51 (50.5%)	50 (49.5%)			
	Severe	8 (44.4%)	10 (55.6%)			
AFP(ng/ml), *n* (%)	≤400	123 (57.2%)	92 (42.8%)	**0.038**	4.32	*χ* ^2^ test
	>400	27 (41.5%)	38 (58.5%)			
Albumin(g/dl), *n* (%)	<3.5	40 (58%)	29 (42%)	0.669	0.18	*χ* ^2^ test
	≥3.5	125 (54.1%)	106 (45.9%)			
Prothrombin time, *n* (%)	≤4	115 (55.3%)	93 (44.7%)	0.329	0.95	*χ* ^2^ test
	>4	43 (48.3%)	46 (51.7%)			
Child-Pugh grade, *n* (%)	A	128 (58.4%)	91 (41.6%)	0.628		Fisher test
	B	12 (57.1%)	9 (42.9%)			
	C	0 (0%)	1 (100%)			
Fibrosis Ishak score, *n* (%)	0	43 (57.3%)	32 (42.7%)	0.710	1.38	*χ* ^2^ test
	½	20 (64.5%)	11 (35.5%)			
	¾	17 (60.7%)	11 (39.3%)			
	5/6	43 (53.1%)	38 (46.9%)			
Vascular invasion, *n* (%)	No	117 (56.2%)	91 (43.8%)	0.344	0.89	*χ* ^2^ test
	Yes	55 (50%)	55 (50%)			
OS event, *n* (%)	Alive	131 (53.7%)	113 (46.3%)	0.065	3.41	*χ* ^2^ test
	Dead	56 (43.1%)	74 (56.9%)			
DSS event, *n* (%)	Alive	158 (55.1%)	129 (44.9%)	**<0.001**	12.66	*χ* ^2^ test
	Dead	25 (31.6%)	54 (68.4%)			
PFS event, *n* (%)	Alive	109 (57.1%)	82 (42.9%)	**0.007**	7.23	*χ* ^2^ test
	Dead	78 (42.6%)	105 (57.4%)			

The values in bold were statistically significant.

### High CEP192 expression was an independent prognostic factor for the overall survival and disease-free survival of HCC

**Figure 3:**
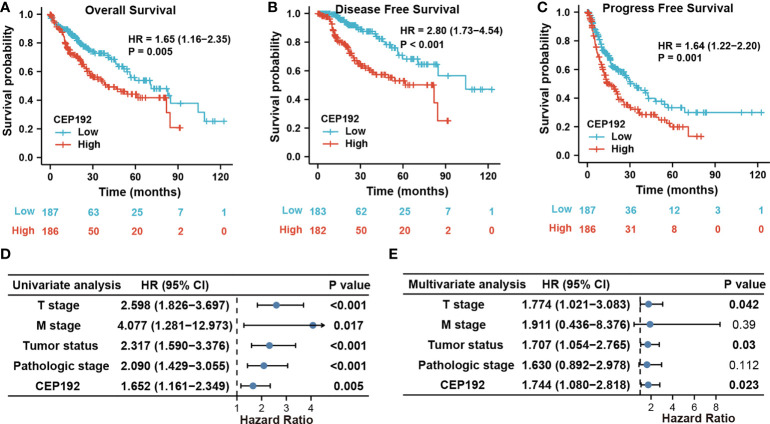
CEP192 expression was correlated with poor survival outcomes in HCC patients. **(A–C)** Kaplan–Meier analysis for overall survival **(A)**, disease-free survival **(B)**, and progression-free survival **(C)** based on high versus low CEP192 expression in TCGA-LIHC samples; Log-rank (Mantel-Cox) test. **(D)** Forest plot showing the univariable Cox model results of T stage, M stage, tumor status, pathologic stage, and CEP192 expression in HCC; Cox proportional hazards regression. **(E)** Forest plot showing the multivariable Cox model results of the five risk factors in **(D)**; Cox proportional hazards regression.

Next, we evaluated the prognostic significance of CEP192 in HCC patients using Kaplan–Meier curves, along with the Cox proportional hazard model. Survival curves illustrated that HCC patients with higher expression of CEP192 had shorter OS, DFS, or PFS than those with lower expression of CEP192 (**Figures 3A** **–C**). Meanwhile, univariate and multivariate Cox regression analyses were carried out to determine the independent prognostic factors for poor survival in HCC patients. Through univariate Cox regression analysis, single variables including high CEP192 mRNA expression, late T stage, M stage, and advanced pathologic stage were significantly associated with poor OS (**Figure 3D**), DFS, and PFS of HCC patients (**Table 2**). Furthermore, variables with a significant difference of less than 0.1 in univariate Cox regression were included for multivariate Cox regression analysis. The result implied that CEP192 expression could serve as an independent prognostic factor for OS (**Figure 3E**, **Table 3**) and DFS in HCC patients (**Table 3**).

**Table 2:** Univariate Cox proportional hazard models for overall survival (OS), disease-free survival (DFS), and progression-free survival (PFS) in HCC patients.

Variables	OS		DFS		PFS	
	HR (95% CI)	*p*-value	HR (95% CI)	*p*-value	HR (95% CI)	*p*-value
**T stage**						
T1	Reference		Reference		Reference	
T2	1.43 (0.90–2.26)	0.129	1.62 (0.87–3.02)	0.129	2.02 (1.41–2.89)	**<0.001**
T3 and T4	2.95 (1.98–4.39)	**<0.001**	4.33 (2.58–7.25)	**<0.001**	2.80 (1.97–3.98)	**<0.001**
N stage						
N0	Reference		Reference		Reference	
N1	2.03 (0.50–8.28)	0.324	3.61 (0.87–14.99)	0.077	1.37 (0.34–5.55)	0.659
**M stage**						
M0	Reference		Reference		Reference	
M1	4.08 (1.28–12.97)	**0.017**	5.17 (1.25–21.43)	**0.024**	3.48 (1.09–11.08)	**0.035**
**Tumor status**						
Tumor free	Reference				Reference	
With tumor	2.32 (1.59–3.38)	**<0.001**			11.34 (7.57–17.00)	**<0.001**
**Pathologic stage**						
Stage I	Reference		Reference		Reference	
Stage II and Stage III and Stage IV	2.09 (1.43–3.06)	**<0.001**	2.91 (1.72–4.93)	**<0.001**	2.28 (1.67–3.12)	**<0.001**
Gender						
Female	Reference		Reference		Reference	
Male	0.79 (0.56–1.13)	0.200	0.81 (0.52–1.28)	0.373	0.98 (0.72–1.34)	0.909
Age						
≤60	Reference		Reference		Reference	
>60	1.21 (0.85–1.71)	0.295	0.85 (0.54–1.32)	0.458	0.96 (0.72–1.28)	0.783
Histologic grade						
G1	Reference		Reference		Reference	
G2 and G3 and G4	1.19 (0.72–1.96)	0.499	1.20 (0.63–2.27)	0.578	1.22 (0.81–1.85)	0.347
AFP (ng/ml)						
≤400	Reference		Reference		Reference	
>400	1.08 (0.66–1.76)	0.772	0.87 (0.45–1.67)	0.668	1.05 (0.70–1.56)	0.832
Vascular invasion						
No	Reference		Reference		Reference	
Yes	1.34 (0.89–2.04)	0.163	1.28 (0.71–2.31)	0.418	1.68 (1.20–2.39)	**0.003**
**CEP192**						
Low	Reference		Reference		Reference	
High	1.65 (1.16–2.35)	**0.005**	2.80 (1.73–4.54)	**<0.001**	1.64 (1.22–2.20)	**<0.001**

The values in bold were statistically significant.

**Table 3:** Multivariate Cox proportional hazard models for overall survival (OS), disease-free survival (DFS), and progression-free survival (PFS) in HCC patients.

Variables	OS		DFS		PFS	
	HR (95% CI)	*p*-value	HR (95% CI)	*p*-value	HR (95% CI)	*p*-value
T stage						
T1	Reference		Reference		Reference	
T2	0.61 (0.08–4.63)	0.633	0.31 (0.04–2.46)	0.266	0.48 (0.06–3.77)	0.488
T3 and T4	1.11 (0.15–8.18)	0.922	0.97 (0.13–7.26)	0.975	0.75 (0.10–5.83)	0.786
M stage						
M0	Reference		Reference		Reference	
M1	1.91(0.44–8.37)	0.391	4.38 (0.95–20.12)	0.058	2.06 (0.60–7.09)	0.254
**Tumor status**						
Tumor free	Reference				Reference	
With tumor	1.70 (1.05–2.75)	**0.032**			14.70 (8.57–25.21)	**<0.001**
Pathologic stage						
Stage I	Reference		Reference		Reference	
Stage II and Stage III and Stage IV	2.62 (0.35–19.95)	0.351	4.63 (0.60–35.87)	0.142	1.71(0.23–12.81)	0.601
**Vascular invasion**						
No					Reference	
Yes					1.66 (1.00–2.76)	**0.048**
**CEP192**						
Low	Reference		Reference		Reference	
High	1.73 (1.07–2.81)	**0.025**	3.25 (1.71–6.17)	**<0.001**	1.70 (0.71–1.61)	0.752

The values in bold were statistically significant.

Based on these five prognostic factors (T stage, M stage, tumor status, pathologic stage, and CEP192), we established a clinically prognostic nomogram for predicting the 1-year, 3-year, and 5-year OS in the TCGA-LIHC cohort (**Figure S2A**). Next, a time-dependent ROC curve was drawn and the AUC was calculated to evaluate the prognostic performance of the nomogram. The AUC scores of the 1-, 3-, and 5-year survival were 0.695, 0.747, and 0.713, respectively, suggesting a high sensitivity and specificity of this nomogram model (**Figure S2B**). Moreover, the discrimination accuracy of the nomogram was assessed with the concordance index (C-index, 0.667, 95% CI: 0.632–0.703), where 1.0 reflected perfect discrimination and 0.5 reflected chance alone. Furthermore, the nomogram calibration curves for 1-, 3-, and 5-year survival probabilities were very close to the ideal 45°C diagonal line, indicating that the nomogram was well calibrated, i.e., the nomogram fit the observed data well (**Figure S2C**). In addition, the DCA demonstrated a superior net benefit and clinical utility of the nomogram for 1-year and 3-year survival than other prognostic factors (**Figures S2D, E**).

### CEP192 was associated with hepatic progenitor cell-driven immunosuppressive ecosystem in liver cancer

**Figure 4:**
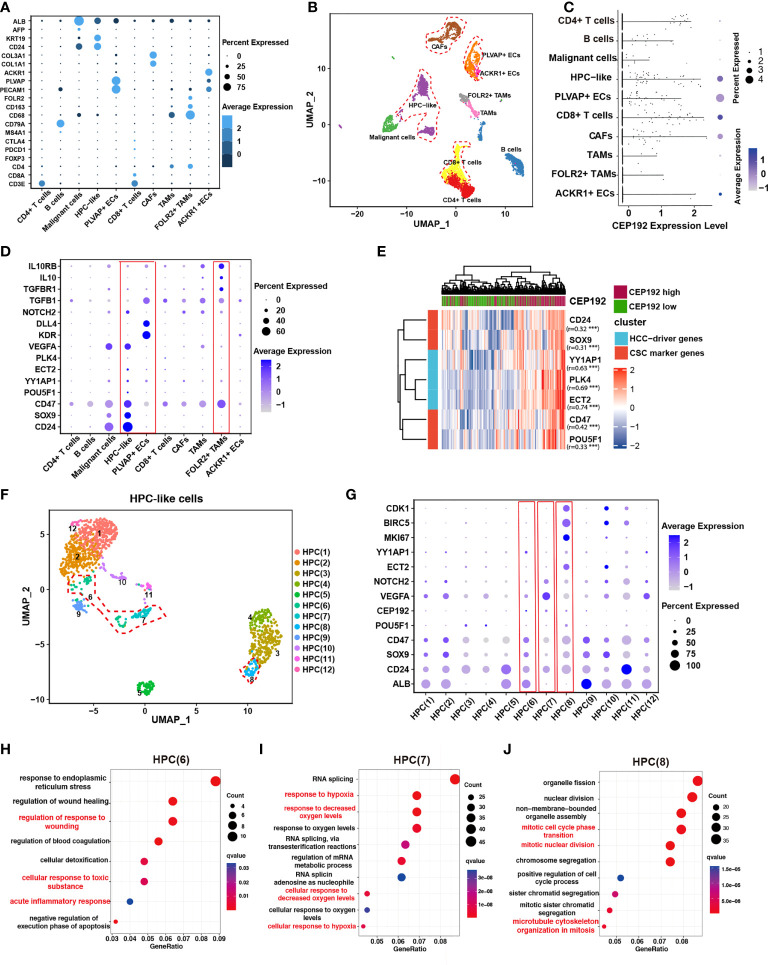
CEP192 was associated with hepatic progenitor cell-driven immunosuppressive ecosystem in liver cancer. **(A)** Dot plot depicting the typically cell-type-specific markers in liver cancer (GSE125449). **(B)** UMAP plot of cell types from 19 tumors (indicated by colors). Cells were annotated as CD4+ T cells, CD8+ T cells, B cells, malignant cells, hepatic progenitor-like cells (HPC-like), endothelial cells that expressed PLVAP genes (PLVAP+ ECs, tumor-specific ECs with fetal-like property), endothelial cells that expressed ACKR1 genes (ACKR1+ ECs), cancer-associated fibroblasts (CAFs), tumor-associated macrophages (TAMs), and TAMs that expressed FOLR2 (FOLR2+ TAMs, fetal-like TAMs with immunosuppressive property) according to cell-type-specific marker genes in **(A)**. **(C)** The expression distribution of CEP192 in liver cancer. **(D)** Expression of gene sets involved in maintaining the immunosuppressive onco-fetal tumor microenvironment *via* the VEGFA/NOTCH signaling in liver cancer. Red boxes highlighted cell clusters that reprogrammed the immunosuppressive onco-fetal ecosystem. **(E)** Heatmap of positive correlation between CEP192 and liver cancer stem cell markers. **(F)** Sub-clustering of HPC-like cells identified 12 cell types. HPC clusters with high CEP192 expression were highlighted in red contours. **(G)** Dot plot of HPC cluster marker genes across HPC-like cells. Red boxes highlighted HPC clusters of high CEP192 expression. **(H–J)** Bubble plot showing the GO-BP enrichment results of HPC(6) **(H)**, HPC(7) **(I)**, and HPC(8) **(J)**.

To elucidate the role of CEP192 during HCC progression, single-cell RNA sequencing (scRNA-seq) of 19 patients with liver cancer (GSE125449) was analyzed to profile CEP192 expression and the liver tumor niche (**Figures 4A, B**). Single-cell transcriptomes of 7,552 cells that passed quality control filtering were obtained, embedded into low-dimensional eigenvector space using PCA on the most variable genes across all cells, and then clustered cell populations with Uniform Manifold Approximation and Projection (UMAP), a recent dimensionality reduction algorithm that is popular in the scRNA-seq community. These cells were mapped to CD4+ T cells, CD8+ T cells, B cells, malignant cells, hepatic progenitor-like cells (HPC-like), endothelial cells that expressed PLVAP genes (PLVAP+ ECs, tumor-specific ECs with fetal-like property), endothelial cells that expressed ACKR1 genes (ACKR1+ ECs), cancer-associated fibroblasts (CAFs), tumor-associated macrophages (TAMs), and TAMs that expressed FOLR2 (FOLR2+ TAMs, fetal-like TAMs with immunosuppressive property) according to cell-type-specific marker genes (**Figures 4A** **, B**). These results are remarkably consistent with previous reports (22, 24). Meanwhile, CEP192 showed a broad expression landscape in most clusters. Moreover, the exact expression level and proportion of CEP192 were visualized in **Figure 4C**, which indicated a greater proportion of cells expressed by CEP192 in HPC-like cells, PLVAP+ EC, CAF, and CD8+ T cells, so that the average CEP192 expression levels in these cells were higher.

Previous studies have suggested that VEGF, secreted from hepatocytes in cell division, could regulate the re-emergence of fetal-like PLVAP+ endothelial cells, which, in turn, reprogramed immunosuppressive fetal-like FOLR2+ TAMs *via* the DLL4/NOTCH2 signaling axis, thereby maintaining an immunosuppressive onco-fetal ecosystem in HCC (24). Consistent with this phenomenon, we found that VEGFA was highly expressed in malignant/HPC-like cells, while PLVAP+ ECs notably expressed its receptor KDR (VEGFR2) (**Figure 4D**), suggesting that these cells may interact within the tumor microenvironment. More importantly, FOLR2+ TAMs significantly expressed typical immunosuppressive genes including TGFB1, TGBR1, IL10, and IL10RB, validating the immunosuppressive role of FOLR2+ TAMs in the HCC tumor niche (**Figure 4D**). In conclusion, our results suggest a nexus between malignant/HPC-like cells, onco-fetal PLVAP+ ECs, and FOLR2+ TAMs in the immunosuppressive tumor ecosystem.

Given the observed expression of CEP192 in HPC-like cells and PLVAP+ ECs, we hypothesized that CEP192 may participate in HPC-driven immunosuppressive niche in liver cancer. This concept was further supported by the positive correlation of CEP192 with characteristic onco-fetal genes, also known as cancer stem cell markers, including CD24, SOX9, CD47, and POU5F1 (OCT4) (**Figure 4E**). More importantly, several validated HCC-driver genes, such as YY1AP1 and ECT2, were highly expressed in HPC-like cells and markedly correlated with CEP192 expression in HCC tissues (**Figure 4E**). Subsequently, the HPC-like cells were classified into 12 subpopulations (**Figure 4F**), and CEP192 was expressed in HPC (6), HPC (7), and HPC (8) (**Figure 4G**). Next, GO enrichment analysis of HPC(6)-specific genes revealed a series of cellular responses to wounding, wound healing, toxic substance, and endoplasmic reticulum stress (**Figure 4H**). Moreover, HPC (6) weakly expressed the hepatocyte-specific gene ALB; therefore, HPC (6) may be hepatocyte precursor cells. Moreover, HPC(7) expressed relatively high levels of CEP192, VEGFA (vascular endothelial growth factor A, the central angiogenic cytokine), and the fetal-like gene CD24 (**Figure 4G**). Meanwhile, HPC(7) exhibited gene signatures in response to hypoxia based on GO term enrichment analysis (**Figure 4I**). These results suggested that HPC(7) represented a pro-angiogenic subpopulation of HPC-like cells. Additionally, GO analysis of differentially expressed genes (DEGs) in cluster HPC(8) revealed strong enrichment of functions related to cell cycle process, cell cycle phase transition, and microtubule cytoskeleton organization in mitosis (**Figure 4J**). This result confirmed the critical role of CEP192 in regulating the cell cycle progression of HPC-like cells. Taken together, CEP192 was expressed in hepatocyte precursor cells, pro-angiogenic VEGFA+ HPC-like cells, and proliferative MKI67+ HPC-like cells.

**Figure 5:**
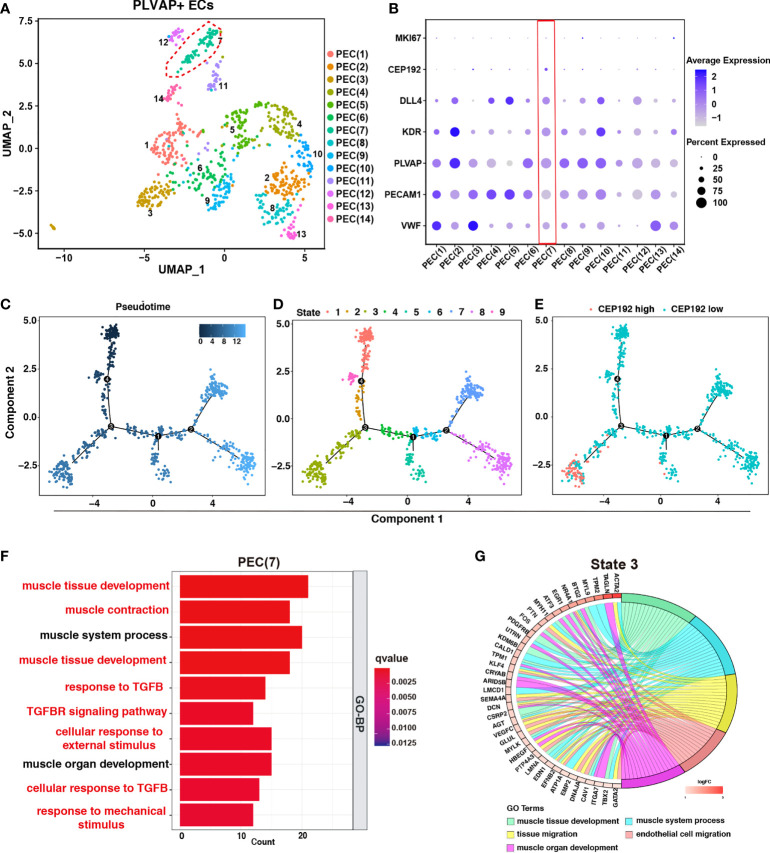
The effects of CEP192 on the function of PLVAP+ ECs. **(A)** UMAP was colored by 14 subpopulations of PLVAP+ ECs. **(B)** Expression of CEP192 and EC-specific marker genes in PLVAP+ EC subpopulations. Red boxes highlighted PLVAP+ EC clusters of high CEP192 expression. **(C–E)** Single-cell trajectory of pseudotime **(C)**, state **(D)**, and CEP192 expression **(E)**. **(F)** Box plot showing the GO-BP enrichment results of PEC(7). **(G)** Chord diagram exhibiting the GO-BP enrichment results of PLVAP+ ECs in state 3.

Next, to assess the effects of CEP192 on the functions of PLVAP+ ECs, we first divided PLVAP+ ECs into 14 subsets *via* dimensionality reduction and examined the expression of CEP192 in each subset (**Figures 5A** **, B**). CEP192 was highly expressed in cluster PEC(7) cells (**Figure 5B**). Pseudotime trajectory modeling was reconstructed using the “Monocle” R package to infer cell state transitions over time. Four main branch points dissected these trajectories into nine distinct state trajectory branches, and CEP192 was highly increased in state 3 (**Figures 5C** **–E**). Functional enrichment analysis revealed that upregulated genes in PEC(7) played roles in muscle tissue development, muscle contraction, and cellular responses to transforming growth factor beta (TGFB), mechanical, and external stimuli (**Figure 5F**). Consistently, the genes upregulated in state 3 were mainly involved in the development of muscle tissue/organ and migration of tissue/endothelial cells (**Figure 5G**). Studies have reported that endothelial cells could be transformed into smooth muscle-like cells in a transforming growth factor beta (TGFβ)-dependent manner (25). Therefore, CEP192 may play a role in the development of endothelium-derived smooth muscle-like cells.

### Correlation analysis of CEP192 expression and tumor microenvironment in HCC

**Figure 6:**
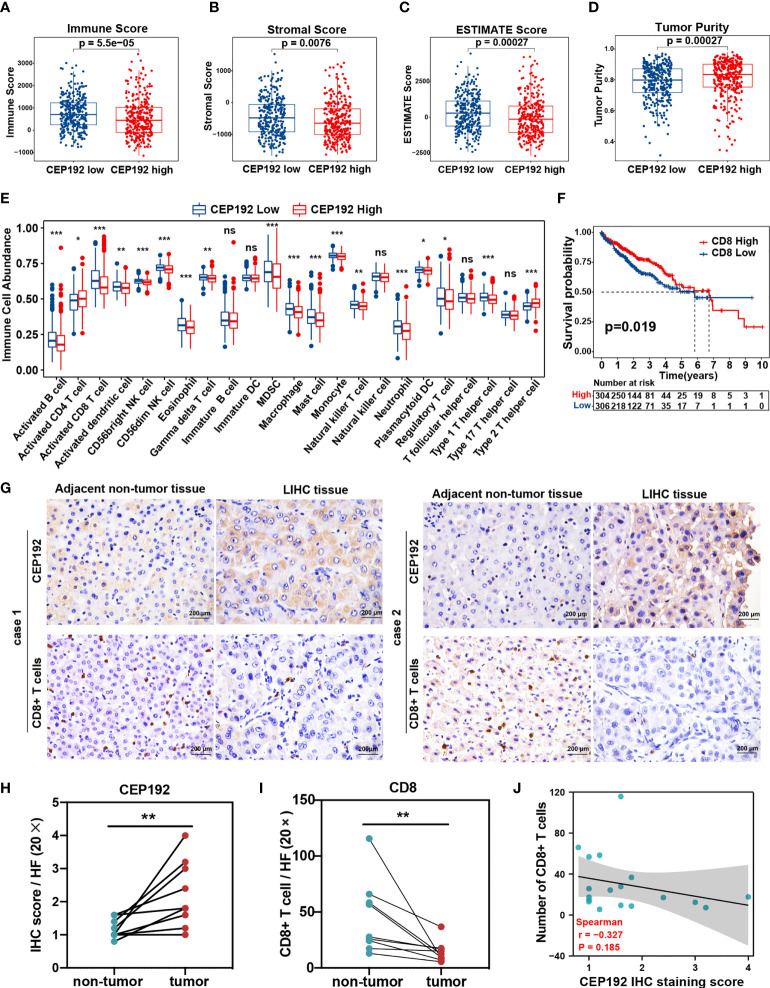
Correlation analysis of CEP192 expression and immune cell infiltration in HCC. **(A–D)** Immune Score **(A)**, Stromal Score **(B)**, ESTIMATE Score **(C)**, and Tumor Purity **(D)** between the CEP192 low (*n* = 306) and CEP192 high (*n* = 305) group based on HCC patients from TCGA-LIHC (*n* = 371) and ICGC-LIRI-JP (*n* = 240) datasets. Mean ± SEM, Mann–Whitney *U* test. **(E)** Distribution of 23 types of immune cells in the CEP192 low (*n* = 306) and high (*n* = 305) groups based on ssGSEA. Mean ± SEM, Mann–Whitney *U* test, *p < 0.05, **p < 0.01, ***p < 0.001. **(F)** Kaplan–Meier curve described the significant survival difference between CD8 high (*n* = 306) and low groups (*n* = 304). Log-rank (Mantel-Cox) test. **(G)** Immunohistochemical staining showing the level of infiltrating CD8+ T cells and CEP192 expression in HCC tumor tissues (*n* = 9) compared with adjacent non-tumor liver tissues (*n* = 9). Scale bar: 200 μm. **(H)** The CEP192 expression levels in **(G)** were quantified with an IHC score using ImageJ (*n* = 9); mean ± SEM, paired *t*-test, **p < 0.01. **(I)** The number of infiltrating CD8+ T cells in **(G)** was counted by a pathologist (*n* = 9); mean ± SEM, paired *t*-test, **p < 0.01. **(J)** Correlation analysis of CEP192 expression and CD8+ T-cell infiltration in **(G)**; Spearman’s rank correlation rho. NS, non significant.

To further investigate the association between CEP192 expression and the tumor microenvironment of HCC, we calculated the immune score, stromal score, ESTIMATE score (that represented the sum of the immune score and stromal score and inferred tumor purity), and tumor purity using the ESTIMATE algorithm in 611 HCC patients downloaded from TCGA (*n* = 371) and ICGC (*n* = 240). The immune score, stromal score, and ESTIMATE score of the CEP192 high group were lower than those in the CEP192 low group (**Figures 6A** **–C**), whereas tumor purity was opposite (**Figure 6D**). Furthermore, we evaluated the infiltration of immune cells in the distinct CEP192 group based on the ssGSEA method and found that the vast majority of immune cells, including innate-immune cells [gamma delta T cells (Tgd), natural killer cells (NK), natural killer T cells (NKT), monocytes, myeloid-derived suppressor cells (MDSCs), macrophages, neutrophils, mast cells, dendritic cells (DCs), and eosinophils] and adaptive-immune cells [B cells, CD8+ T cells, type 1 T helper cells (Th1), and regulatory T cells (Treg)], were significantly decreased in the CEP192 high group of HCC (**Figure 6E**). In contrast, CD4+ T cells and type 2 T helper cells (Th2) were enriched in the CEP192 high group (**Figure 6E**). More importantly, patients with high CD8+ T-cell infiltration had lower CEP192 expression and better OS compared to those with low CD8+ T cell infiltration (**Figures 6F**, **S3A**). Moreover, the infiltrating CD8+ T cells in liver cancer tissues decreased with the stage (**Figure S3B**). Also, immunohistochemical analysis showed an increase in CEP192 expression accompanied by a decrease in the number of infiltrating CD8+ T cells in tumor tissues compared with that in non-tumor tissues (**Figures 6G** **–I**). Though not as dramatic, a tendency of negative correlation between CEP192 and CD8 was observed in the IHC staining samples (**Figure 6J**).

**Figure 7:**
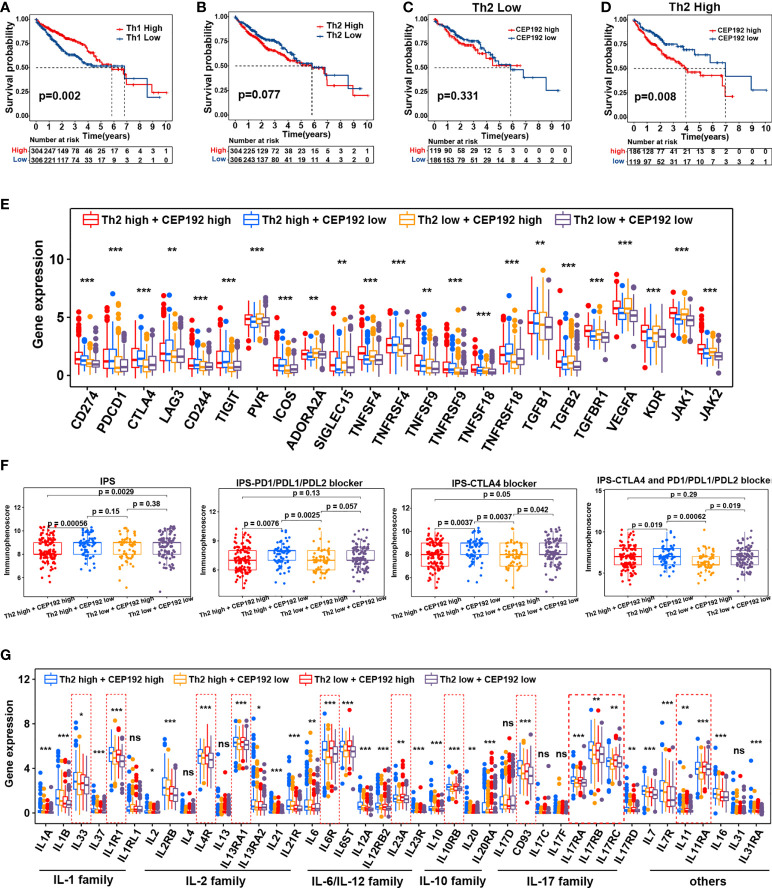
Correlation analysis of CEP192 expression with immunotherapy response and inflammatory cytokine levels. **(A)** Kaplan–Meier curves showing the survival difference in the patients with low versus high levels of Th1 cells (Th1 Low, *n* = 306; Th1 High, *n* = 304). Log-rank (Mantel-Cox) test. **(B)** Kaplan–Meier curve showing the survival difference between Th2 high (*n* = 304) and Th2 low groups (*n* = 306). Log-rank (Mantel-Cox) test. **(C)** Kaplan–Meier curve showing the survival difference between CP192 high (*n* = 119) and CP192 low (*n* = 186) groups in HCC patients with low infiltration of Th2 cells. Log-rank (Mantel-Cox) test. **(D)** Kaplan–Meier curve showing the survival difference between CP192 high (*n* = 186) and CP192 low (*n* = 119) groups in HCC patients with high infiltration of Th2 cells. Log-rank (Mantel-Cox) test. **(E)** Expression of 23 immune checkpoints in the Th2 high + CEP192 high (*n* = 186), Th2 high + CEP192 low (*n* = 119), Th2 low + CEP192 high (*n* = 119), and Th2 low + CEP192 low (*n* = 186) groups. Mean ± SEM, Kruskal–Wallis test, **p < 0.01, ***p < 0.001. **(F)** Comparison of IPS, IPS-PD1/PD-L1/PD-L2 blocker, IPS-CTLA4, and IPS-CTLA4 + PD1/PD-L1/PD-L2 blocker scores of Th2 high + CEP192 high (*n* = 127), Th2 high + CEP192 low (*n* = 71), Th2 low + CEP192 high (*n* = 62), and Th2 low + CEP192 low (*n* = 110) groups in HCC patients from TCGA-LIHC. Mean ± SEM, Kruskal–Wallis test. **(G)** Expression of interleukins and associated receptors in the Th2 high + CEP192 high (*n* = 186), Th2 high + CEP192 low (*n* = 119), Th2 low + CEP192 high (*n* = 119), and Th2 low + CEP192 low (*n* = 186) groups based on HCC patients from TCGA-LIHC (*n* = 371) and ICGC-LIRI-JP (*n* = 240) datasets. Mean ± SEM, Kruskal–Wallis test, *p < 0.05, **p < 0.01, ***p < 0.001. NS, non significant.

Contrary to the event of tumor-infiltrating CD8+ T cells, patients with high CD4+ T-cell infiltration had worse OS compared with those with low CD4+ T-cell infiltration (**Figure S3C**). Activated CD4+ T cells differentiate into different effector cells, including Th1, Th2, TFH, Th9, and Th17, based on the expression of costimulatory molecules, antigen presentation, and cytokine profiles (26). Here, the CEP192 mRNA level was increased in tumors with a high infiltrate of CD4+ T cells or Th2 cells, and decreased in tumors with a high infiltrate of Th1 cells or Tregs (**Figure S3A**). Although the median OS in patients with high tumor-infiltrating Tregs was shorter than that in patients with tumor-infiltrating Tregs, there was no statistically significant difference in survival outcomes between the two groups (**Figure S3D**). In contrast, patients with low Th1 cell infiltration exhibited worse OS and higher T classification (**Figures 7A**, **S3E**). Moreover, a high infiltration of Th2 cells correlated with decreased OS and advanced disease of patients with HCC (**Figures 7B**, **S3F**). Furthermore, survival analyses revealed a synergistic effect of CEP192 expression and Th2 cell infiltration on adverse clinical outcomes in HCC patients (**Figures 7C, D**). Additionally, expression levels of multiple critical immunosuppressive molecules, such as CD274, PDCD1, CTLA4, LAG3, CD244, TIGIT, ICOS, TNFSF9, TNFRSF9, TNFSF18, and TNFRSF18, were found to be higher in the Th2 high group than in the Th2 low group (**Figure 7E**). More importantly, CEP192 expression was also positively correlated with the expression of multiple immunosuppressive genes including CD274, PVR, ADORA2A, SIGLEC15, TNFSF4, TGFB2, TGFBR1, VEGFA, KDR, JAK1, and JAK2 (**Figure 7E**). Taken together, CEP192 might help establish an immunosuppressive microenvironment through interactions with Th2 cells in the HCC tumor niche.

Considering the positive correlation between the immune checkpoint and CEP192, we further tested the relevance of CEP192 to ICIs. Both TMB and IPS have been proven to be effective in predicting the response of ICIs (27). Therefore, the correlation of CEP192 with TMB and IPS was conducted. There was no significant association between TMB and CEP192 level (**Figure S3G**). The immune checkpoint molecules programmed cell death protein 1 (PD-1), programmed cell death 1 ligand 1 (PD-L1), and cytotoxic T-lymphocyte-associated protein 4 (CTLA4) were targets of current clinically relevant immunotherapies. Thus, the scores of IPS, IPS-PD1/PD-L1/PD-L2 blocker, IPS-CTLA4, and IPS-CTLA4 + PD1/PD-L1/PD-L2 blocker were calculated to predict response to immunotherapy with CTLA-4 and PD-1 blockers. As expected, the scores of IPS-PD1/PD-L1/PD-L2 blocker, IPS-CTLA4, and IPS-CTLA4 + PD1/PD-L1/PD-L2 blocker were all lower in the CEP192 high group than in the CEP192 low group (**Figure 7F**), suggesting less immunogenicity on ICIs in the CEP192 high group. These data indicated that HCC tumors with low CEP192 expression were more likely to respond to PD-L1, PD-1, and CTLA4 blocker immunotherapy.

Interleukins and associated receptors are the main effectors in regulating cross-talk between immune and non-immune cells in the tumor milieu. We next explored the expression difference of these cytokines between cancer patients with low and high CEP192 expression. Some members of the IL-1 family (IL1R1), IL-2 family (IL4R, IL13RA1), IL-6 family (IL6R and IL23A), IL-10 family (IL10RB), IL-17 family (IL17RA, IL17RB, and IL17RC), and others (IL11, IL11RA) were upregulated in the CEP192 high group compared with the CEP192 low group (**Figure 7G**). Furthermore, scRNA-seq was used to predict the ligand–receptor interactions of interleukins among T cells, malignant cells, HPC-like cells, ECs, TAMs, and CAFs. IL11 and IL17D were highly expressed in HPC-like cells, whereas IL11RA and CD93 (receptors for IL11 and IL17D, respectively) were mainly expressed in PLVAP+ ECs. In turn, PLVAP ECs may interact with TAMs *via* IL6–IL6R, with CAFs *via* IL33–IL1RL1, and with themselves or CD8+ T cells *via* the IL12A–IL12R axis. Beyond, TAMs and CAFs may interact through the IL1B–IL1R1 and IL6–IL6R axes. Furthermore, CD4+ T cells may activate the immune response of TAMs through IL13–IL13R and may activate the immune response in self or CD8+ T cells through the IL23A–IL23R axis (**Figure S3H**). Therefore, these results suggested that CD4+ T cells, CD8+ T cells, ECs, TAMs, CAF malignant cells, and HPC-like cells may interact closely through the ligand–receptor signaling axes of interleukins to maintain an immunosuppressive microenvironment in the HCC tumor niche.

### CEP192 affected HCC cell proliferation by regulating cell cycle

**Figure 8:**
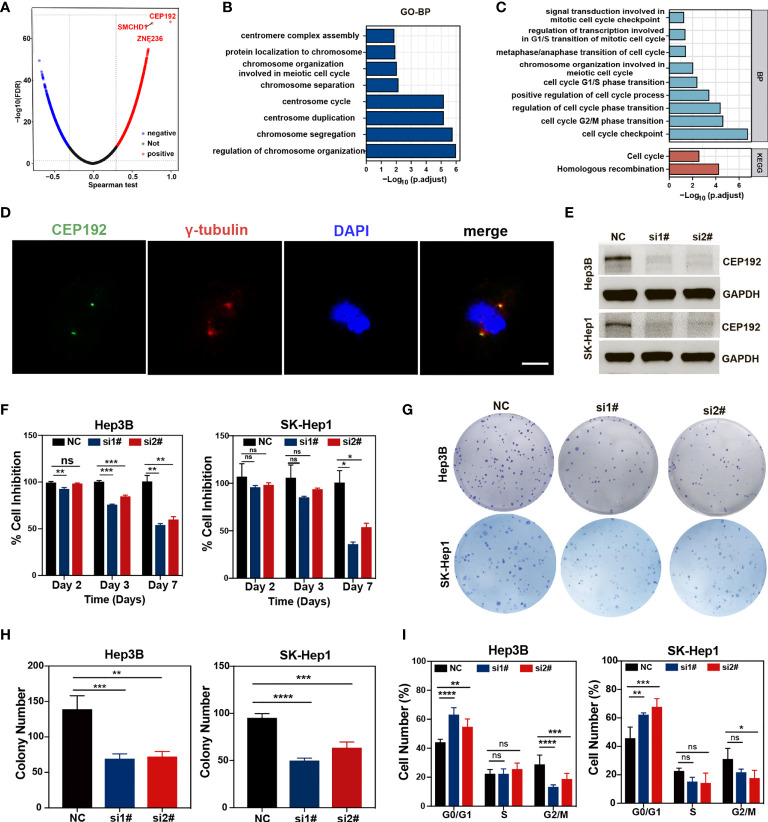
CEP192 was involved in cell cycle regulation. **(A)** Volcano plot showing the genes in the TCGA-LIHC dataset that were positively and negatively related to CEP192. **(B, C)** Biological processes (BP) **(B)** and Kyoto Encyclopedia of Genes and Genomes (KEGG) **(C)** pathway enrichment analysis based on CEP192 correlated genes with Spearman correlation coefficients (SCC) > 0.5. **(D)** Immunofluorescence staining showing the co-localization of CEP192 with centrosome marker γ-tubulin in SK-Hep1 cells. Scale bar: 10 μm. **(E)** CEP192 knockdown efficiency was assessed by Western blotting in Hep3B and SK-Hep1 cell lines on day 3 after transfection with specific CEP192 siRNAs. **(F)** Cell proliferation ability was evaluated by MTT in Hep3B and SK-Hep1 cell lines on day 2, day 3, and day 7 from transfection; mean ± SD, SD represents three independent experiments (*n* = 3), two-way ANOVA, *p < 0.05, **p < 0.01, ***p < 0.001. **(G, H)** Cell self-renewal and colony formation ability of Hep3B and SK-Hep1 cells were determined 2 weeks after transfection with CEP192 siRNAs; mean ± SD, SD represents three independent experiments (*n* = 3), one-way ANOVA, **p < 0.01, ***p < 0.001, ****p < 0.001. **(I)** Flow cytometry was applied to detect the cell cycle distribution of Hep3B and SK-Hep1 cells; mean ± SD, SD represents three independent experiments (*n* = 3), two-way ANOVA, *p < 0.05, **p < 0.01, ***p < 0.001, ****p < 0.001. NS, non significant.

To gain a comprehensive insight into the biological function of CEP192 in HCC, the LinkFinder module of the LinkedOmics website was used to calculate the Spearman correlation coefficients (SCC) between CEP192 and other genes in TCGA-LIHC samples. As shown in the volcano plot (**Figure 8A**), 9,278 genes (red dots) correlated positively with CEP192, and 4,505 genes (blue dots) correlated negatively with CEP192. Furthermore, Gene Ontology (GO) term, Kyoto Encyclopedia of Genes and Genomes (KEGG) pathway, and gene set enrichment analyses (GSEAs) were performed to annotate the functions of CEP192 correlated genes with SCC > 0.5. This result revealed that CEP192 correlated genes may engage in the regulation of cell cycle progression, such as centrosome cycle, chromosome segregation, G1/S phase transition, G2/M phase transition, and metaphase/anaphase transition (**Figures 8B** **, C**, **Figure S4A, B**). Additionally, CEP192 was localized to the centrosome based on its colocalization with the centrosome marker, γ-tubulin, which was consistent with the previous report (**Figure 8D**).

To further test the regulatory effect of CEP192 on the cell cycle, we knocked down endogenous CEP192 using CEP192-targeted siRNA sequences. The efficiency of siRNA knockdown was assessed using quantitative polymerase chain reaction (qPCR) and Western blotting (WB). Knockdown efficiency of 50% was observed at the mRNA level (**Figure S4C**), which corresponded with a knockdown effect at the protein level, as determined by WB (**Figure 8E**). We then examined the effect of CEP192 silencing on tumor cell proliferation and self-renewal using MTT and tumor colony formation assay. MTT results indicated that CEP192 silencing inhibited the proliferation of Hep3B and SK-Hep1 cells in a time-dependent manner, with ~50% inhibition efficiency at 7 days post-silencing (**Figure 8F**). Similarly, CEP192 silencing in Hep3B and SK-Hep1 cells markedly decreased the colony number (**Figures 8G** **, H**). More importantly, in contrast to the control clones, characterized by tightly connected cells, the CEP192-silencing clones were very loosely connected (**Figure S4D**). Furthermore, propidium iodide (PI) staining in combination with flow cytometry was used to determine the distribution of cells over the different phases of the cell cycle at 7 days post-silencing. Compared to cells with scramble siRNA, both Hep3B and SK-Hep1 cells with CEP192 siRNAs exhibited a marked different distribution of cell cycle, with a significant increase in the proportion of cells in the G0/G1 phase and a concomitant decrease in the proportion of cells in the G2/M phase (**Figures 8I**, **S4E**). Consistent with the previously presented KEGG, GO-BP, and GSEA, these results provided evidence that CEP192 regulated HCC cell proliferation by promoting cell cycle progression.

## Discussion

CEP192 (centrosomal protein 192) was first cloned in 2000 by Nagase et al. using sequencing (28), which is composed of a PapD-like domain with two sites for serine phosphorylation and a homologous sequence to *C. elegans* Spd2, a protein that was considered to be indispensable for centriole duplication (29). Centrosome amplification has been extensively reported in both hematological malignancies and solid tumors (30). Moreover, CEP192 is highly expressed in the fetal liver but lowly expressed in the normal adult liver (28). Thus, an oncogenic role was proposed for CEP192 in HCC. Our study for the first time suggested that CEP192 was upregulated in HCC tissues and correlated significantly with tumor progression and adverse prognosis. Upregulation of CEP192 was further validated to be an independent prognostic factor for poor outcomes in HCC.

A small subset in the tumor population, termed cancer stem cells (CSC) or tumor-initiating cells, also known as hepatic progenitor-like cells (HPC-like cells) in liver cancer, possess stem-like properties including limitless proliferation, self-renewal, and multilineage differentiation, and it has been characterized in a variety of cancerous tissues. Accumulating evidence indicates that CSCs are emerging as critical mediators of therapy resistance including chemotherapy, targeted therapies, and immunotherapy (31). Here, based on scRNA-seq data, we found that CEP192 was notably expressed in HPC-like cells but very low in ALB+ malignant cells, an expression pattern consistent with mouse liver development. Moreover, CEP192 was also discovered to be positively correlated with cancer stem cell markers including CD24, SOX9, CD47, and POU5F1 (OCT4). Subsequently, we identified 3 hepatic progenitor-like subpopulations with high CEP192 expression in liver cancer tissues, which exhibited robust proliferative potential and response to hypoxia, wounding, wound healing, toxic substance, and endoplasmic reticulum stress. Therefore, CEP192 may be a potential biomarker for cancer stem-like cells in liver cancer.

Sharma et al. found that VEGFA secreted by proliferative hepatocytes induced the re-emergence of fetal-like PLVAP+ endothelial cells *via* directly regulating PLVAP expression, which, in turn, reprogrammed immunosuppressive fetal-like FOLR2+ tumor-associated macrophages (TAMs) *via* the DLL4/NOTCH2 signaling axis, thereby maintaining an immunosuppressive onco-fetal ecosystem in liver cancer (24). In our study, except for malignant cells (ALB+ hepatocyte), VEGFA was markedly expressed in CEP192-expressing HPC-like cells, and KDR (VEGFA receptor) was highly expressed in fetal-like PLVAP+ endothelial cells. In turn, PLVAP+ endothelial cells notably expressed DLL4, while FOLR2+ TAMs significantly expressed NOTCH2 (DLL4 receptor) as well as immune-suppressing genes (TGFB1, TGBR1, IL10, and IL10RB). Therefore, CEP192 may participate in HPC-driven immunosuppressive niche in liver cancer. Then, we evaluated the association between CEP192 expression and the tumor microenvironment in 611 HCC patients using ESTIMATE (32) and the ssGSEA algorithm (33), both of which were popular in the immune infiltration analysis community. Most immune cells were significantly decreased in the CEP192 high group than in the CEP192 low group, whereas type 2 T helper cell (Th2) and M2 macrophage were enriched in the CEP192 high group. Th2 as well as Th1, Th17, T follicular helper (TFH), and regulatory T (Treg) cells differentiate from naïve CD4+ T cells upon activation with cytokines and inducible transcription factors (34). Though there have been contradictory reports on the role of these T helper cells in cancer development, in general, Th1 exerts antitumor immunity (35), while Th2 seems to induce an immunosuppressive microenvironment (36). Here, we found that liver cancer patients with low levels of Th1 were associated with advanced disease and poor prognosis. Conversely, liver cancer patients with high levels of Th2 were correlated with advanced stage, poor prognosis, and higher expression of immune-inhibitory molecules, which confirmed the immunosuppressive role of Th2 in liver cancer.

Cytokines released into the tumor tissues were pivotal to shape the tumor milieu and facilitate tumor growth. We found that patients with high CEP192 levels showed a different cytokine profile compared with patients with high CEP192 levels. Of all cytokines increased in tumors with high CEP192 expression, IL11, a robust inducer of STAT3 activation, was reported to be increased in HCC tumors and play a critical role in postsurgical recurrence (37). We found that IL11 was specifically expressed in HPC-like cells and may interact with fetal PLVAP+ ECs and malignant cells through its receptor IL11RA. Then, the PLVAP+ ECs may express IL6 to activate TAMs, which, in turn, maintain an immunosuppressive tumor microenvironment *via* the IL10–IL10RB axis. IL6 and IL10 were known to be key pro-tumor cytokines that exerts multiple functions in the development of liver tumors, including promoting proliferation, EMT, angiogenesis, anti-apoptosis, and immune surveillance evasion (38, 39). IL13 is an immunomodulatory cytokine primarily produced by activated Th2 cells and may be involved in inhibiting the production of pro-inflammatory cytokines and chemokines (40). We found that IL13 was expressed in CD4+ T cells, while its receptor IL13RA1 was mainly expressed in TAMs, PLVAP+ ECs, and HPC-like cells, suggesting that CD4+ T cells may have an effect on TAMs, PLVAP+ ECs, and HPC-like cells. Referring to existing studies, our finding demonstrated close cytokine ligand–receptor interactions within the tumor niche to regulate HCC immune evasion and HCC progression, and that the expression of those cytokines was highly correlated with CEP192.

Centrosome, the best-known microtubule organizing centers (MTOCs), is recognized as the central signaling hub of the cell cycle, and CEP192 is believed to be the most critical centrosomal component in mitosis, because of its indispensable role in centriole duplication, pericentriolar material (PCM) recruitment, microtubule nucleation, centrosome maturation, and mitotic spindle assembly (41). In our research, bioinformatics prediction demonstrated that CEP192 was mainly located at the centrosome and may play a crucial role in the centrosome cycle, chromosome segregation, and cell cycle. Subsequent immunofluorescence staining validated its colocalization with the centrosome marker, γ-tubulin. Silencing CEP192 using siRNAs increased the proportion of cells in the G0/G1 phase and decreased the cell populations in the G2/M phase of the cell cycle, thereby preventing cell cycle progression and arresting tumor cell growth. This result confirmed a critical role of CEP192 in the cell cycle, which was consistent with previous reports (29, 42). Furthermore, we also observed that cep192-silenced cells exhibited elongated and loosely connected morphologies. Consistent with our study, Sharp et al. reported that depletion of CEP192 increased the cell axial ratio (length/width) and reduced cell migration (43). Possible reasons behind this proposed by Sharp et al. are a decrease in radial centrosomal microtubules that tend to maintain cell shape and an increase in directional Golgi apparatus or cytosolic microtubules that are involved in cellular elongation and polarization (43).

It is noteworthy that centrosome proteins, such as Aurora A, PLK1, and PLK4, have received substantial attention as biomarkers and drug targets. Thus, small-molecule kinase inhibitors (KIs) that target Aurora A, PLK1, and PLK4, respectively, have attracted intense interest, leading to dozens of KIs being designed, synthesized, and tested in preclinical and clinical settings, with some even being used in clinical trials (44–46). However, KIs of Aurora A, PLK1, and PLK4 as monotherapies in previous clinical trials were not satisfactory; enough evidence has suggested that KIs can significantly enhance the therapeutic efficacy of several established treatment regimens, including radiotherapies, chemotherapies, targeted therapies, and immunotherapies (45). Recently, the PLK4 inhibitor, CFI-400945, has been shown to potentially improve the efficiency of PD-1 blockade in a late-stage mouse HCC model. Notably, CFI-400945-mediated cell cycle arrest induced senescence and a senescence-associated secretory phenotype (SASP), which recruited anti-tumorigenic immune cells and converted immunologically “cold” tumor microenvironments to “inflamed” or “hot” microenvironments (14). Given the critical roles of CEP192 in the cell cycle, it is worth further exploring the impacts of CEP192 silencing on senescence and SASP factors, which may provide evidence for CEP192 as a therapeutic target to remodel the tumor microenvironment in HCC tissues.

Currently, most available KIs under clinical investigation are ATP-competitive inhibitors that target the ATP-binding pocket of the kinase domain (KD), with certain limitations, including poor specificity due to the high structural similarity among family members, high risk of certain adverse effects, and acquired resistance mutations frequently arise at the ATP-binding sites (45). Moreover, clinical trials of these KIs as single agents failed to meet therapeutic expectations, with marginal clinical efficacies in both hematologic malignancies and solid cancers (46). Consequently, short interfering RNAs (siRNA) targeting Aurora A and PLK1, respectively, have been proposed as alternative treatment options with enhanced selectivity and reduced side effects (47–50). Considering these observations, chemically modified or nanoparticle-formulated siRNA or aptamer designed to target CEP192 may be potential therapeutic strategies for tumors, especially for HCC.

In conclusion, we first uncovered a potential onco-immunological role of CEP192 in HCC. First, increased CEP192 expression in HCC was associated with tumor progression and predicted poor prognosis. Second, CEP192 was notably expressed in three hepatic progenitor-like subsets, which were characterized by robust proliferative potential and response to hypoxia, wounding, wound healing, toxic substance, and endoplasmic reticulum stress. Third, CEP192 was highly associated with a variety of pro-tumor cytokine ligand–receptor axes, including IL11–IL11RA, IL6–IL6R, and IL13–IL13RA1, which may promote interactions between HPC-like cells, PLVAP+ ECs, TAMs, and CD4+ T cells to drive immunosuppression. Accordingly, CEP192 expression was closely associated with an immunosuppressive tumor microenvironment and low IPS, making it a potential predictor of response to ICIs. Last but not least, CEP192 promoted cell proliferation and cell cycle progress by taking part in the regulation of centrosome function. Taken together, CEP192 may present a promising prognostic indicator and therapeutic target for HCC patients. However, much remains unknown about the underlying mechanisms of CEP192 to modulate the immunosuppressive microenvironment in HCC. Further research is needed to detect the tumor-infiltrating immune cell populations and cytokines in an orthotopic mouse HCC model with CEP192 inhibition, which will provide definitive evidence for the immunosuppressive role of CEP192 in HCC.

## Data availability statement

The original contributions presented in the study are included in the article/**Supplementary Material**. Further inquiries can be directed to the corresponding author.

## Ethics statement

This study was approved by the Academic Committee of No. 2 Affiliated Hospital, Guangzhou Medical University, Guangzhou, China, and written informed consent was obtained from all donors. The investigation was conducted according to the Declaration of Helsinki principles. In addition, all the datasets were collected from the publishing literature, so all written informed consent was obtained from the individual(s) for the publication of any potentially identifiable images or data included in this article.

## Author contributions

Conception and design: YLL and JC. Foundation support: YLL and JC. Acquisition and analysis of data: YLL, WL, YC, ZH, HZ, XK, MW, ML, QQL, QYL, WT, QH, YL, QY, CM, ZM, JW, and HS. Interpretation of data: YLL, KP, MH, and JC. Drafting the manuscript and revising for submission quality: YLL, XK, MW, and JC. Study supervision: JC. All authors contributed to the article and approved the submitted version.

## Funding

This work was supported by grants from National Natural Science Foundation of China, the Natural Science Foundation of China (81172537, 81272900, 81772828), Scientific and Technological Project of Guangdong Province (2016A020216028), the China Postdoctoral Science Fund (2018M643045), the Basic and Applied Basic Research Project of Guangzhou Science and Technology Bureau (202201011003), and the Ph.D. Start-up Fund of Second Affiliated Hospital of Guangzhou Medical University.

## Acknowledgments

We appreciate researchers who have developed and maintained public databases such as TCGA, GEO, TISIDB, TIMER, TCIA, and LinkedOmics, which will accelerate the understanding and treatment of human cancer.

## Conflict of interest

The authors declare that the research was conducted in the absence of any commercial or financial relationships that could be construed as a potential conflict of interest.

## Publisher’s note

All claims expressed in this article are solely those of the authors and do not necessarily represent those of their affiliated organizations, or those of the publisher, the editors and the reviewers. Any product that may be evaluated in this article, or claim that may be made by its manufacturer, is not guaranteed or endorsed by the publisher.

## Glossary

**Table:**

HCC	Hepatocellular carcinoma
AFP	alpha-fetoprotein
ICIs	Immune checkpoint inhibitors
OS	Overall survival
PFS	Progression-free survival
ORR	Objective response rates
TMB	Tumor mutation burden
PLK4	Polo-like kinase 4
NAFLD	Non-alcoholic fatty liver disease
mRNA	Messenger RNA
TCGA	The Cancer Genome Atlas
GEO	Gene Expression Omnibus Datasets
DSS	Disease-free survival
PFS	Progression-free survival
HR	Hazard ratio
ROC	Receiver operating characteristic
DCA	Decision curve analysis
IHC	Immunohistochemistry
DAB	3,3′-diaminobenzidine
FBS	Fetal bovine serum
NCBI	National Center for Biotechnology Information
DMSO	Dimethyl sulfoxide
PI	Propidium iodide
TMB	Tumor-mutation burden
IPS	Immunophenoscore
TCIA	The Cancer Immunome Atlas
CI	Confidence intervals
AUC	Area under receiver operating characteristics curve
SCC	Spearman correlation coefficients
GO	Gene Ontology
BP	Biological process
GSEA	Gene set enrichment analyses
qPCR	Quantitative polymerase chain reaction
TISIDB	Immune System Interaction Database
TIL	Tumor-infiltrating lymphocytes
Tgd	gamma delta T cells
NK	Natural killer cells
NKT	Natural killer T cells
CD8+ T cells	CD8+ cytotoxic T cells
DC	dendritic cells
Th1	Type 1 T helper cells
MDSC	Myeloid-derived suppressor cells
CAF	Cancer associated fibroblast
Treg	Regulatory T cell
CD4+ Th2	Type 2 T helper cells
CTLA-4	Cytotoxic T lymphocyte antigen-4
PD-1	Programmed cell death protein 1
PD-L1	Programmed death ligand-1
TKIs	Targeted therapies
CEP192	Centrosomal protein 192
MTOCs	Microtubule organizing centers
PCM	Pericentriolar material
PLK1	Polo-like kinase 1
γ-TuRC	γ-tubulin ring complex
CKAP5	Cytoskeleton-associated protein 5
PLK4	Polo-like kinase 4
Kis	Kinase inhibitors
KD	Kinase domain
siRNA	Short interfering RNA
SASP	Senescence-associated secretory phenotype
TNBC	Triple‐negative breast cancer
MHC	Major histocompatibility complex
