# Supplementary material for: CEP192 is a novel prognostic marker and correlates with the immune microenvironment in hepatocellular carcinoma
Source: Front Immunol. 2022 Sep 27;13:950884. doi: 10.3389/fimmu.2022.950884 (PMC9551108; doi:10.3389/fimmu.2022.950884)
Supplement: Supplementary file 1 [file DataSheet_1.pdf]

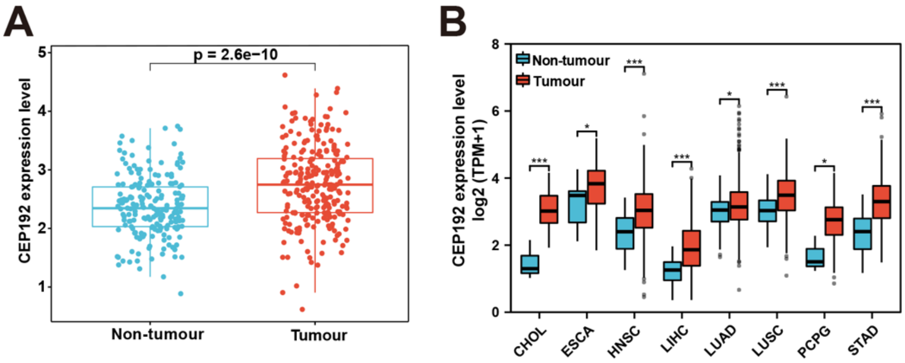

Supplementary Figure 1. CEP192 expression profiles in adjacent non-tumour tissues and tumours. (A) Differences of CEP192 expression between liver tumours (n = 240) and adjacent non-tumour samples (n = 197) from the International Cancer Genome Consortium (ICGC) dataset. Mean ± SEM, Mann-Whitney U test. (B) Transcription levels of CEP192 in TCGA datasets of multiple cancers (T) compared with adjacent non-tumour tissues (N). Mean ± SEM, Mann-Whitney U test, *P < 0.05, ***P < 0.001. CHOL, Cholangiocarcinoma (N, n = 9; T, n = 36); ESCA, Esophageal carcinoma (N, n = 11; T, n = 162); HNSC, Head and Neck squamous cell carcinoma (N, n = 44; T, n = 502); LIHC, Liver hepatocellular carcinoma (N, n = 50; T, n = 374); LUAD, Lung adenocarcinoma (N, n = 59; T, n = 535); LUSC, Lung squamous cell carcinoma (N, n = 49; T, n = 502); PCPG, Pheochromocytoma and Paraganglioma (N, n = 3; T, n = 183); STAD, Stomach adenocarcinoma (N, n = 32; T, n = 375).

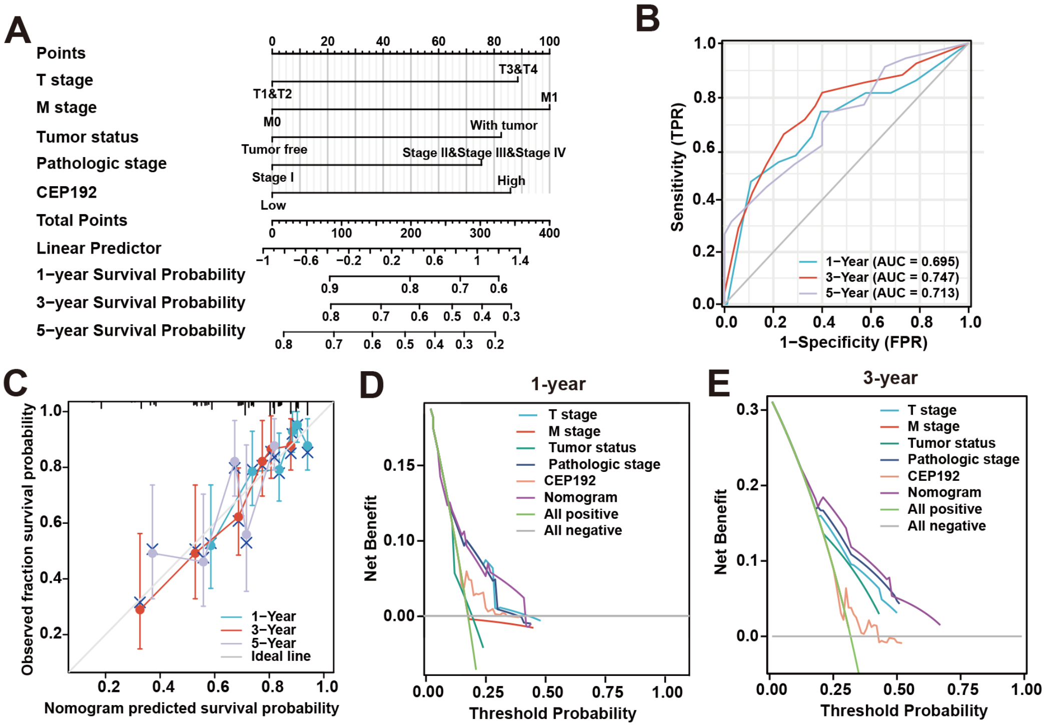

Supplementary Figure 2. Construction and evaluation of prognostic nomogram model. (A) Nomogram was constructed based on T stage, M stage, tumour status, pathologic stage, and CEP192 expression to predict the 1-year, 3-year, and 5-year overall survival in the TCGA-LIHC cohort; Cox proportional hazards regression. (B) ROC curves showing the sensitivity and specificity of the nomogram to predict the overall survival outcome of HCC patients in the TCGA dataset. (C) Calibration plots illustrating the accuracy of the nomogram for predicting 1, 3, and 5-year survival probabilities. (D-E) Decision curve demonstrating the net benefit and clinical utility of the nomogram for 1-year (D) and 3-year (E) survival.

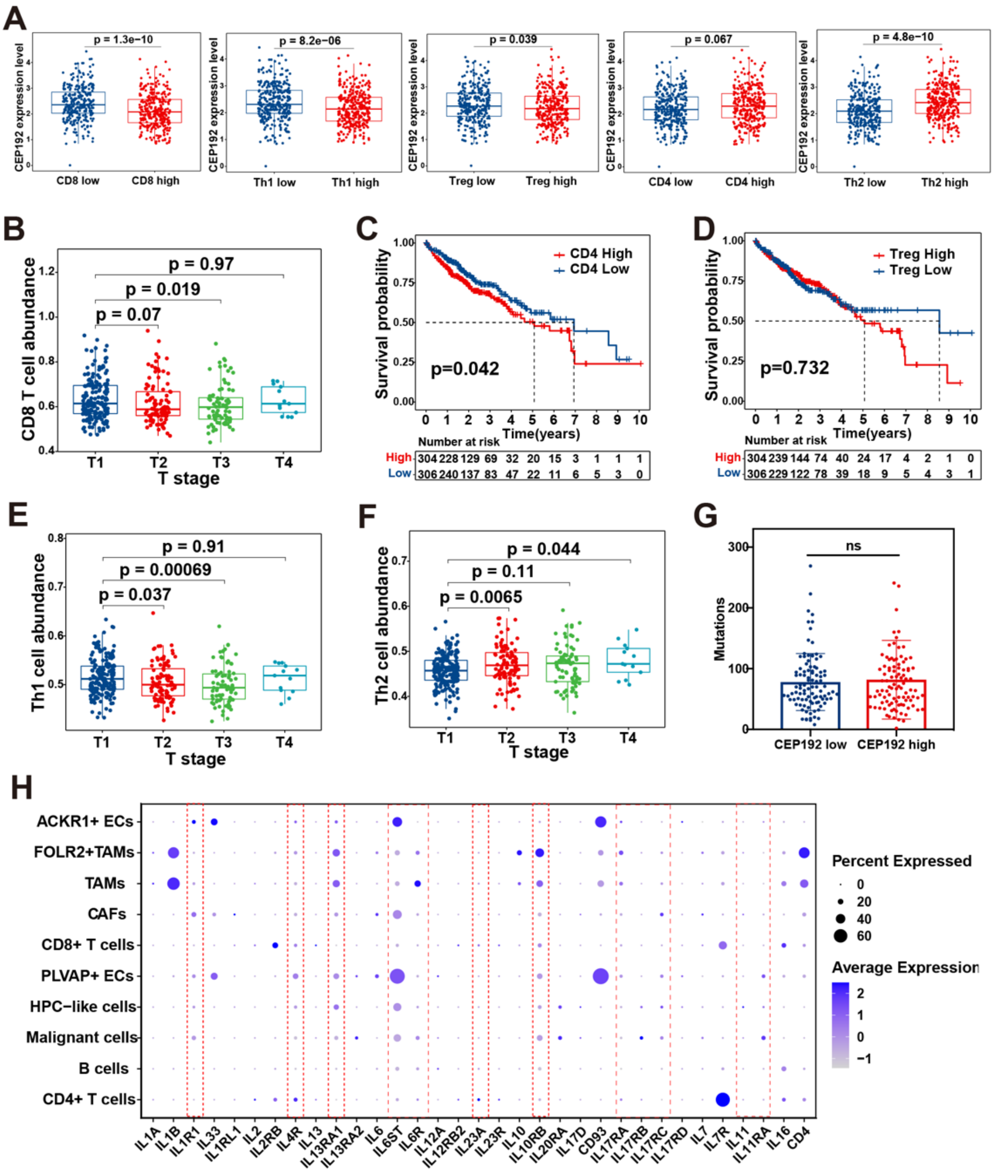

Supplementary Figure 3. Correlation analysis of CEP192 expression and immune cells infiltration in HCC. (A) CEP192 expression difference in the patients with low (n = 306) versus high (n = 305) infiltration levels of different subtypes of T cells. Mean ± SEM, Mann-Whitney U test. (B) CD8+ T cell infiltration levels of TCGA-LIHC samples at different T stages of liver cancer (T1, n = 185; T2, n = 95; T3, n = 81; T4, n = 13); Mean ± SEM, Kruskal-Wallis test. (C) Kaplan-Meier curves showing the survival difference in the patients with low versus high levels of CD4+ T cell (CD4 Low, n = 306; CD4 High, n = 304). Log-rank (Mantel-Cox) test. (D) Kaplan-Meier curves showing the survival difference in the patients with low versus

high levels of Tregs (Treg low, n = 306; Treg high, n = 304). Log-rank (Mantel-Cox) test. (E) Th1 infiltration levels of TCGA-LIHC samples at different T stages of liver cancer (T1, n = 185; T2, n = 95; T3, n = 81; T4, n = 13); Mean ± SEM, Kruskal-Wallis test. (F) Th2 infiltration levels of TCGA-LIHC samples at different T stages of liver cancer (T1, n = 185; T2, n = 95; T3, n = 81; T4, n = 13); Mean ± SEM, Kruskal-Wallis test. (G) Tumour mutation burden in TCGA-LIHC patients with high CEP192 expression (n = 98) and low CEP192 expression (n = 97). (H) The expression distribution of CEP192-related cytokines and their receptors within the HCC tumour niche.

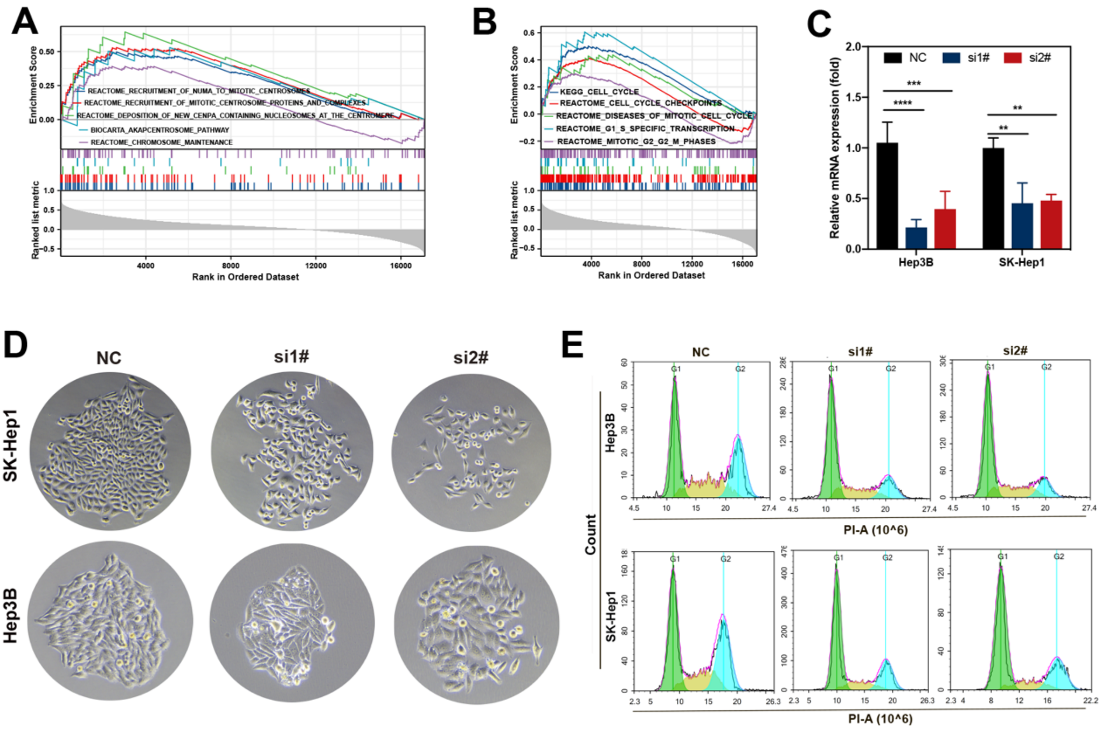

Supplementary Figure 4. CEP192 was associated with cell cycle pathways in HCC. (A-B) Gene set enrichment analysis (GSEA) on CEP192 correlated genes with P < 0.05. (C) CEP192 knockdown efficiency was assessed by qPCR in Hep3B and SK-Hep1 cell lines on day 3 after transfection with specific CEP192 siRNAs. (D) Bright-field imaging showing the colony morphology of Hep3B and SK-Hep1 cells

transfected with specific CEP192 siRNAs for two weeks. Scale bar: 300 μm. (E) Cell cycle distributions were determined in Hep3B and SK-Hep1 cells using flow cytometry.

Supplementary Table 1. HCC datasets information

Dataset	Non-tumour (*n*)	Tumour (*n*)	Dataset Link
GSE14520	220	225	https://www.ncbi.nlm.nih.gov/geo/query/acc.cgi?acc=GSE14520
GSE45267	39	48	https://www.ncbi.nlm.nih.gov/geo/query/acc.cgi?acc=GSE45267
GSE121248	37	70	https://www.ncbi.nlm.nih.gov/geo/query/acc.cgi?acc=GSE121248
GSE36376	193	240	https://www.ncbi.nlm.nih.gov/geo/query/acc.cgi?acc=GSE36376
GSE76427	52	115	https://www.ncbi.nlm.nih.gov/geo/query/acc.cgi?acc=GSE76427
GSE65372	15	39	https://www.ncbi.nlm.nih.gov/geo/query/acc.cgi?acc=GSE65372
TCGA-LIHC	50	371	(https://portal.gdc.cancer.gov/)
ICGC	197	240	https://ocg.cancer.gov/e-newsletter-issue/issue-11/international-cancer-genome-consortium
